# High-resolution in situ transcriptomics of *Pseudomonas aeruginosa* unveils genotype independent patho-phenotypes in cystic fibrosis lungs

**DOI:** 10.1038/s41467-018-05944-5

**Published:** 2018-08-27

**Authors:** Elio Rossi, Marilena Falcone, Søren Molin, Helle Krogh Johansen

**Affiliations:** 1 Department of Clinical Microbiology, Rigshospitalet, DK-2100 Copenhagen Ø, Denmark; 2 Novo Nordisk Foundation Center for Biosustainability, Technical University of Denmark, DK-2800 Kgs. Lyngby, Denmark; 3 Department of Clinical Medicine, Faculty of Health and Medical Sciences, University of Copenhagen, DK-2200 Copenhagen N, Denmark

## Abstract

Life-long bacterial infections in cystic fibrosis (CF) airways constitute an excellent model both for persistent infections and for microbial adaptive evolution in complex dynamic environments. Using high-resolution transcriptomics applied on CF sputum, we profile transcriptional phenotypes of *Pseudomonas aeruginosa* populations in patho-physiological conditions. Here we show that the soft-core genome of genetically distinct populations, while maintaining transcriptional flexibility, shares a common expression program tied to the lungs environment. We identify genetically independent traits defining *P*. *aeruginosa* physiology in vivo, documenting the connection between several previously identified mutations in CF isolates and some of the convergent phenotypes known to develop in later stages of the infection. In addition, our data highlight to what extent this organism can exploit its extensive repertoire of physiological pathways to acclimate to a new niche and suggest how alternative nutrients produced in the lungs may be utilized in unexpected metabolic contexts.

## Abstract (web summary)

How genetic adaptation and phenotypic acclimation are interrelated and allow *Pseudomonas aeruginosa* to persist in cystic fibrosis lungs is poorly understood. Here, Rossi et al. use high-resolution transcriptomics on expectorates to link phenotypic conservation to ecological flexibility and persistence.

## Introduction

Viscous secretions in the lungs of cystic fibrosis (CF) patients create a favorable environment for microbial infections. *Pseudomonas aeruginosa* is one of the most frequently encountered bacteria in CF airways, and despite antibiotic treatment the infections most often become chronic. Aggressive antibiotic treatment, however, is able to control progression and exacerbations of the infection^1^ allowing patients to survive for decades. During this long time of infection, complex selective forces drive *P*. *aeruginosa* adaptation through genetic modifications such as single-nucleotide polymorphisms^2^ and various genetic rearrangements^3,4^. These adaptive mechanisms transform environmental clones into descendant lineages capable of persisting in the CF airways. To date, the adaptive processes of long-term lung survival have been described through whole-genome sequencing (WGS) of longitudinal collections of *P*. *aeruginosa* CF isolates, and identification of a small number of sequential patho-adaptive mutations conserved in genetically distant *P*. *aeruginosa* populations^2,5–7^. However, since CF isolates display a high genetic and phenotypic variability^8^, genetic studies are limited in their ability to predict how the observed genetic differences are translated into better adapted phenotypes in the actual environment, in which the bacterial physiology plays a critical role in determining the final outcome^9^. Thus, independent clones having gone through independent evolutionary trajectories can achieve similar expression programs in the same environment^10^, and it is not uncommon that conserved patho-adaptive mutations do not display the expected phenotypic properties^2^.

Although different studies have gained knowledge about how genetic adaptation contributes to reshape bacterial gene expression in *P*. *aeruginosa* CF isolates^5,11^, the difficulties in recreation of the conditions characterizing CF lungs^12,13^ have prevented establishment of a complete and realistic overview of how genetically distant lineages behave in the lungs. In particular, it is unknown to what extent evolutionarily distinct lineages adopt a similar gene expression program that reflects common phenotypic traits contributing to persistence in patho-physiological conditions. To address these issues, we have developed a meta-transcriptomics investigation strategy for the total RNA content in CF sputum, analyzing at the single-gene level the transcriptional profile of *P*. *aeruginosa* communities in their real environment as indicators of their in vivo phenotypes. We successfully applied this strategy to a cohort of CF patients infected by different *P*. *aeruginosa* lineages, which have adapted for more than 200,000 generations to the CF lung environment, thus representing a late stage of the evolutionary process. We show that, despite a long independent evolutionary history constrained in the lungs of specific patients, a common end point transcriptional program specifically connected to the CF lung environment can be identified. In particular, we identify lineage-independent gene expression profiles affected by the patho-physiological conditions in the CF lungs, which are associated with responses to stress (oxidative, antibiotics, and osmotic) and physiological acclimation, and find evidence for changed utilization of multiple redundant and/or alternative nutrient acquisition systems, suggesting that metabolic rewiring may contribute to *P*. *aeruginosa* ecological flexibility and persistence in the lungs.

## Results

### *Pseudomonas aeruginosa* high-resolution in vivo transcriptome

**Fig. 1:**
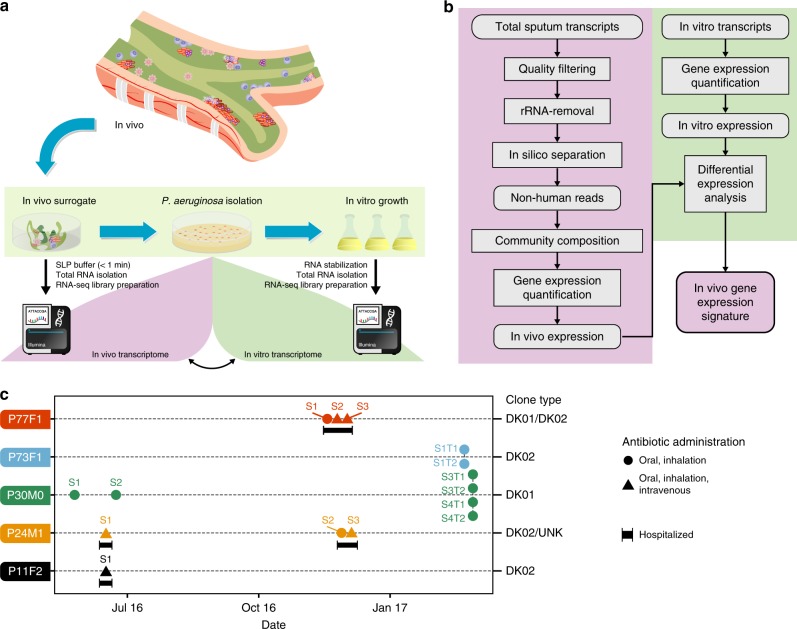
Experimental design and patients included in the study. **a** Schematic representation of the experimental design. In the thick mucus layer, bacterial communities thrive in a harsh environment. To capture the best representation of in vivo transcription, sputum samples were collected directly from adult CF patients followed at the Copenhagen Cystic Fibrosis Clinic at Rigshospitalet and nucleic acid content was stabilized in less than 1 min using sputum pre-lysis and preservation buffer (SLP buffer), followed by total RNA isolation, RNA-seq library preparation, and sequencing (for a complete description see Methods). From the same or a second sputum sample, we isolated, for some patients, more than ten single *P. aeruginosa* clones and studied their gene expression in laboratory condition (in vitro). **b** Simplified schematic representation of the in silico analysis workflow. Total reads were quality-filtered and any rRNA contaminant removed. Human reads were separated by mapping high-quality reads directly on human GRCh38 genome. Reads not assignable to human genome were used to evaluate microbial community composition and to assess in vivo *P. aeruginosa* gene expression. Transcription deriving from in vitro cultures of *P. aeruginosa* were used as a reference for identifying differentially gene expressed in vivo. **c** Overview of longitudinally collected samples. Clone types colonizing each patient are reported. Assignment of clone type was obtained by whole genome sequences from isolates obtained during this study or from previous isolates. A generic indication of antibiotic treatment and intravenous administration is provided (for a complete overview see Supplementary Table 1)

In order to investigate in vivo gene expression of *P*. *aeruginosa* populations infecting CF lungs, we performed RNA-sequencing directly on 12 CF sputum samples obtained from 5 chronically infected patients (Fig. 1 and Supplementary Table 1). For three samples (P24M1_S1, P30M0_S1, and P11F2), we sequenced total RNA without depletion of ribosomal RNA in order to evaluate the feasibility of the technique and the percentage of reads deriving from the human transcriptome. Stable rRNA accounted for nearly 70% of the total sequences recovered, and human transcripts dominated the non-ribosomal RNA pool (ca. 95%) (Supplementary Table 2). Non-human RNA, including bacterial RNA, represented on average only 5% of the total reads (Supplementary Table 2). In order to obtain a high-resolution analysis of the *P*. *aeruginosa* transcriptome, we depleted rRNA from all subsequent samples and generated ~180 million reads per sample. Informative non-rRNA transcript numbers increased three times with ~150 million non-rRNA reads per sample of which 4% represented non-human reads (Supplementary Fig. 1 and Supplementary Table 2). Although all samples yielded enough reads assignable to the *P*. *aeruginosa* genome sufficient for a high-resolution transcriptome, we excluded those not depleted from ribosomal RNA. In this way we avoided additional bias deriving from a different processing procedure.

Analysis of the relative representation of the major transcriptionally active microbial genera in each sputum sample (Supplementary Note 1 and Supplementary Figs. 2 and 9) reveled a low complexity microbiome, typical of a late stage of the infection. As expected, *Pseudomonas* represented the dominant genus in all patients (Supplementary Fig. 2), while the additional identified genera have previously been associated with old chronically infected patients^14–17^, and included both Gram-positive (*Streptococcus*, *Rothia*, *Staphylococcus*, and *Granulicatella*) and Gram-negative bacteria (*Riemerella*, *Stenotrophomonas*, *Porphyromonas*, and *Veillonella*) (Supplementary Fig. 2); no obvious bias towards one of the two bacterial classes was observed.

The presence of other bacterial species did not interfere with correct assignment of reads to *P*. *aeruginosa* (Supplementary Note 2 and Supplementary Fig. 1,0). For all samples, we were able to uniquely map between 1 and 10 millions reads to the *P*. *aeruginosa* genome (Supplementary Table 3), providing sufficient coverage to reliably quantify and compare transcripts abundance across samples^18^. On average, we detected 87% (range 72–95%, median 90%) of the annotated coding sequences (CDS), with a positive correlation between the number of detectable transcripts and the number of generated reads (Supplementary Fig. 3).

The established workflow was found to be reproducible (Supplementary Note 3), introducing only negligible variations in technical replicates of *P*. *aeruginosa* transcriptomes. Indeed, normalized read counts showed a strong correlation (Pearson’s correlation coefficient >0.99; Supplementary Fig. 4) between replicates deriving from a single sputum sample that was split in two, each part being processed independently. Similarly, we observed a high correlation (*r* = 0.99) when we compared the transcriptional profiles of *P*. *aeruginosa* obtained from two independent expectorates collected and analyzed on the same day from a single patient (P30M0_S3 and P30M0_S4) (Supplementary Fig. 4). The correlation was high between the two expectorates despite a great difference in the biological diversity (Supplementary Fig. 2), which might derive from the uneven distribution of bacteria in the sputum or by the formation of heterogeneous aggregates^19,20^. Thus, independent of population size and structural distribution of the bacteria in separate sputum samples, the transcriptomic variation within the dominant *P*. *aeruginosa* population can be considered extremely low within the same patient, and each sputum sample being a good predictor of the transcriptional activity in the lungs at a given time point consistent with reports in other lung pathogens^21^.

### Soft-core genome as basis for inter-clonal comparisons

Two patients of this investigation were infected by single *P*. *aeruginosa* clone types belonging to the DK01 (P30M0) and the DK02 clone type (P73F1), respectively, and one patient (P77F1) by a mixture of these two clones, as suggested from the analysis of whole genome sequencing (WGS) data on clones isolated from the collected expectorates and from previously published data^22^. Patient P24M1 was infected with DK01 in addition to a second previously unidentified lineage (UNK) (Supplementary Table 1). Overall, representative clones obtained from different patients, but belonging to the same clone type, were separated on average by >5000 genetic variations (Supplementary Table 4), suggesting that the analyzed transcriptional profiles always originated from clones with a long independent evolutionary history and with a strong distinct genetic background.

**Fig. 2:**
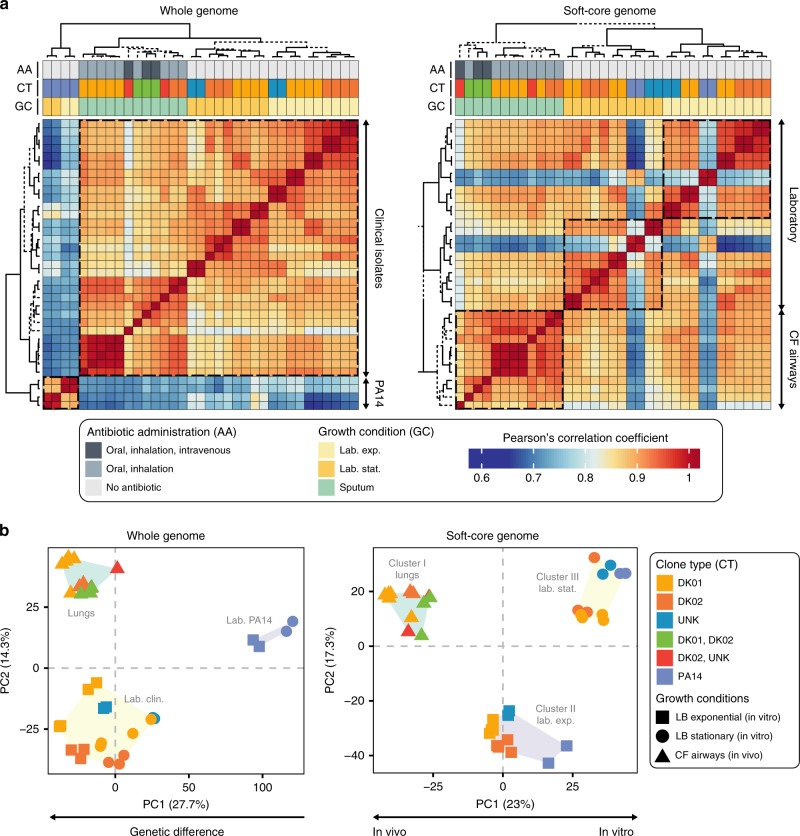
Core genome analysis reduces genetic variability uncovering a shared transcriptional program. **a** Gene expression correlation expressed as Pearson’s correlation coefficient (*r*) and visualized as heat map of expression profiles deriving from the transcriptional activity of the whole genome (left panel, *n* = 5976 CDS) or soft-core genome (right panel, CDS conserved in 95% of the strains considered; *n* = 5102 CDS). Row and column clustering is based on results from pvclust analysis. Major significant clusters are highlighted by dashed rectangles, and solid lines in the side dendrograms. Dashed lines in dendrograms represent branches with AU values <95% and thus not considered significantly supported by data. **b** Cluster refinement by principal component analysis (PCA) and group identification based on k-means clustering on PCA data considering gene expression from the whole genome (left panel) or only coding sequences conserved in the soft-core genome of *P. aeruginosa* (right panel). Clusters identified by k-means analysis are labeled depending on major features of samples included in the group. For both analysis, rLog-normalized counts were used as representation of gene expression (see Methods)

At first, to determine similarities between gene expression profiles of populations growing in the lung environment, we used an unsupervised approach on gene expression deriving from the whole genome (*n* = 5976 CDS). We compared the in vivo transcriptomes with those originating from in vitro cultures of the reference strain PA14 and of five clinical strains isolated from the expectorates used for in vivo RNA-seq (Supplementary Table 1 and Supplementary Fig. 5). We used Pearson correlation combined with hierarchical clustering (HCA) computing the uncertainty in clustering via multiscale bootstrap resampling. Independent of the origin and the growth conditions, two significant clusters corresponding to transcriptomes expressed from the reference strain PA14 and clinical isolates were identified (Fig. 2a and Supplementary Fig. 6), revealing a strong effect of genetic diversity on the analysis; indeed, genes expressed in one strain, might not be expressed in other samples due to genetic variation affecting the correlation measurement^23^. Consistently, in samples with the lowest correlation coefficients we observed the strong presence of outliers represented by non-conserved genes (accessory genes, Supplementary Fig. 7). Genetic diversity also dominated group separation when we used principal component analysis (PCA) and k-means clustering on normalized counts (Fig. 2b).

The impact of genetic diversity can be reduced by restricting the analysis to genes conserved in the species core genomes, with the additional effect of strongly limiting the number of genes under study. In an attempt to provide the best resolution possible while coping with genetic differences, we decided to analyze expression of 5102 (85% of PA14 CDS) genes constituting the soft-core genome (genes conserved in 95% of *P*. *aeruginosa* genomes, Supplementary Data 1 and Methods). Restriction of the analysis to the soft-core genome strongly reduced the presence of outliers in comparisons between genetically distant populations, improving the reliability of the correlation measurement (Supplementary Fig. 7). Thus, HCA showed that separation based on the genetic background was less distinct, while other features, such as growth phases for in vitro samples, and intravenous antibiotic administration for sputum samples, markedly contributed to the formation of the clusters (Fig. 2a). Similarly, PCA analysis reflected these changes separating the transcriptional profiles based on their respective environments and growth state rather than their genetic background (Fig. 2b). We obtained the same results when we considered expression of genes from the far more restrictive core genome of the species (*n* = 2319 CDS) (Supplementary Fig. 7 and Supplementary Fig. 8), indicating that further reducing the number of genes analyzed would not provide any significant benefits to the analysis. Therefore, we focused our analysis on expression of genes belonging to the soft-core genome.

Based on hierarchical clustering and PCA, the data show the formation of three distinct groups of expression: profiles representing in vivo communities (Cluster I, lungs), others representing in vitro cultures in exponential phase (Cluster II, lab. exp.) and stationary phase (Cluster III, lab. stat.) (Fig. 2b). Interestingly, the clusters grouped irrespective of their genetic background and evolutionary history. For example, the reference strain PA14 does not represent a separate group (Fig. 2b), but instead shows transcriptional patterns closely related to clinical isolates growing in vitro, suggesting that expression originating from soft-core genes is driven by conserved signals reflecting growth in a given environment. Moreover, clinical isolates grown in vitro clustered separately from the related in vivo transcriptional profiles, suggesting that clinical strains that evolved in CF lungs maintain a certain degree of gene expression plasticity, through which they achieve similar transcriptional patterns as the genetically distinct PA14 strain. Similarly, in vivo samples grouped together despite differences in genetic background, evolutionary history, patient, co-infecting microbes, and antibiotic treatment (Fig. 2, Supplementary Fig. 2 and Supplementary Table 1). This suggest, as seen in laboratory evolutionary experiments, that genotypes descending from independent evolutionary trajectories can disclose related phenotypic traits reflecting the growth conditions^10^.

### Conserved transcriptional program in multiple clone types

**Fig. 3:**
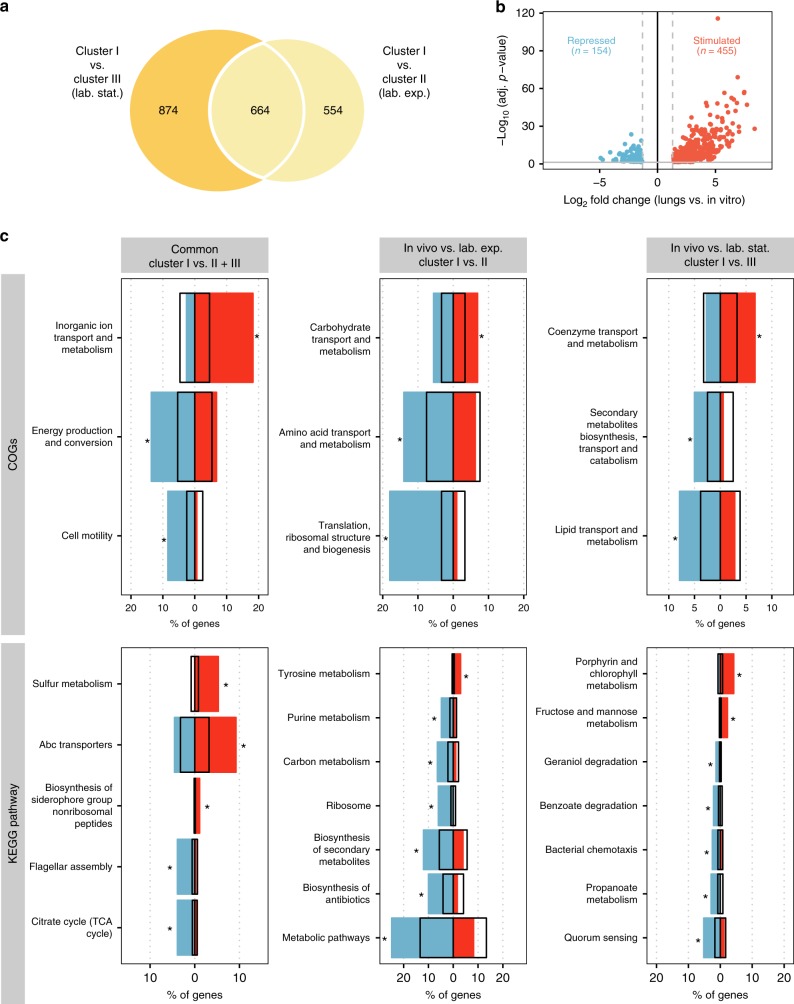
Differentially expressed genes regulated by in vivo conditions and their functional classification. **a** Venn diagram of total differentially regulated genes in the CF expectorates (Cluster I) compared to strains grown in laboratory conditions in exponential (Cluster I vs. Cluster II) and stationary phase (Cluster I vs. Cluster III). Shared genes in the center are highlighted by a white outline. **b** Volcano plot showing the magnitude of the differential gene expression shared between Cluster I vs. Cluster II and Cluster I vs. Cluster III comparisons. Each dot represents one coding sequence with detectable expression in both conditions. Thresholds for defining a gene significantly differentially expressed (log_2_(FoldChange) ≥ |1.3|, adj. *p* value ≤0.05) are shown as dashed and solid lines, respectively. Red dots: genes consistently induced by in vivo conditions. Blue dots: genes consistently repressed by in vivo conditions. **c** Distribution of differentially expressed genes both common and unique to the comparisons Cluster I vs. II and Cluster I vs. III is based on COGs and KEGG pathway classification systems. Gene association for each category was obtained from Pseudomonas.com database. In each plot, the percentage of genes significantly upregulated (red bars on the right) or downregulated (blue bars on the left) associated to each functional category is reported. Asterisks denote functional categories significantly enriched (adjusted *p* value ≤0.05, hypergeometric test after Bonferroni correction). Open black bars represent the proportion of the entire genome in the specific category

Clustering analysis suggested that despite genetic differences, *P*. *aeruginosa* in CF airways had similar transcriptional phenotypes distinct from gene expression of the same bacteria grown in laboratory condition, either in exponential or in stationary phase. To characterize the transcriptional changes defining the *P*. *aeruginosa* physiology when growing in the mucus layer, we compared in vivo (Cluster I, lungs in Fig. 2b) and in vitro transcriptomes, in particular Cluster I vs. Cluster II (lab. exp.) and Cluster III (lab. stat.). We identified 554 and 874 differentially expressed genes when comparing Cluster I vs. Cluster II and Cluster I vs. III, respectively, while 664 genes were expressed similarly in the two comparisons (Fig. 3a and Supplementary Data 2). Gene enrichment analysis based on COGs and KEGG pathway classification systems (Fig. 3c) suggests that expression of genes involved in metabolic pathways (such as carbon and purine metabolism), translation (in particular ribosome biogenesis), amino acid transport and metabolism (with the exception of tyrosine metabolism), and biosynthesis of secondary metabolites are significantly reduced in vivo, when compared with cells growing in exponential phase in laboratory conditions. In contrast, genes belonging to the class of carbohydrate transport and metabolism are significantly stimulated. When compared to cells in stationary phase, in vivo populations had lower expression of genes involved in quorum sensing, chemotaxis, propanoate metabolism, benzoate and geraniol degradation, lipid transport and metabolism, and secondary metabolites transport and metabolism. Genes involved in coenzyme transport and metabolism, in particular in porphyrin metabolism, and those connected with fructose and mannose were increased in vivo when compared to stationary cells in laboratory conditions. Finally, functional enrichment of genes shared between the two comparisons indicated that in in vivo populations transcription of genes involved in inorganic ion transport and metabolism, in particular iron and sulfur uptake, and in ABC transporters was significantly higher. Expression of genes belonging to energy production and conservation, cell motility and flagellar assembly, and TCA cycle functional classes was significantly lower in vivo than in cells growing in vitro in exponential or stationary phases. Thus, in vivo gene expression profiles constitute a unique physiological state different from what is usually defined as exponential or stationary phase in laboratory conditions. This suggests transcriptional acclimation in the CF lung promoting a low-energy, low-growth, non-motile physiological state.

Since the high-level resolution of our approach provided a possibility to analyze environmentally driven changes in detail, we further dissected the contribution of each differentially expressed gene common to all comparisons (Fig. 3a, b) and analyzed how distinct transcriptional processes involved in bacterial stress responses and cell metabolism might be correlated with in vivo conditions.

### Transcriptional acclimation to CF airways stresses

In infected lungs, high levels of reactive (RNS) and oxidative (ROS) agents are present as a result of the host immune system^24^. RNS and ROS cause DNA damage, and consistently we observed upregulation of the DNA-damage-inducible regulator LexA and of several genes under its control (Supplementary Data 2). In vivo responses to RNS species are mainly driven by an increased expression of NO reductase and flavo-hemoglobin protein Fhp (Supplementary Data 2). Similarly, expression of genes encoding ROS scavenging enzymes (*ahpB, ahpC, ahpF*, *katA*, *katB*, *sodM*, *ohr*, and glutathione peroxidases), proteins involved in redox balance maintenance (LsfA, trxB2, PA14_27520, PA14_09950, PA14_32590, and PA14_32595), ROS-insensitive variants of metabolic enzymes (*fumC1*), and homologs to the *E*. *coli* MsrQP system (PA14_62100 and PA14_62110) implicated in repairing periplasmic-oxidized protein^25^ were upregulated (Supplementary Data 2).

An additional source of stress is represented by the continuous antibiotic treatments. In addition to genetically acquired drug resistance, environmentally driven expression of specific and generic mechanisms contributes to establish phenotypically tolerant physiological states, which are thought to be a main determinant for treatment failure. Despite different antibiotic treatments (Supplementary Table 1), we observed a convergent induction in expression of specific efflux pumps and transporters (*mexE* and *qacH*/*emrE*) (Supplementary Data 2), involved in resistance to virtually all antibiotics administered to the patients (Supplementary Table 1)^26–28^. Likewise, expression of the ß-lactamase encoding *ampC* gene and the *ampC*-regulator AmpDh3, and of CzcRS^29^, were higher in vivo contributing to ß-lactam resistance (Supplementary Data 2). Reduction of *pmrB* and *colR* genes involved in resistance to the widely used polymyxin drug class^24,30^, and induction of the aminoglycoside inactivating enzyme, AphA^31^ (Supplementary Data 2) might, as well, contribute to fine-tuning the resistance to these two antibiotic classes.

Finally, the thick dehydrated mucus layer imposes osmotic stress on *P*. *aeruginosa*^12^. In the lung, accumulation of osmo-protectants, in particular glycine-betaine (GB)^32^ obtained from degradation of phosphatidyl choline and sphingomyelin lipids-derived choline^33^ and L-carnitine, seems to represent the major strategy to respond to the high salt concentration. Indeed, high-level expression of genes responsible for choline and L-carnitine degradation to GB (*opuCD*, *betA*, *betB*, and *cdhC*) and of the transcriptional repressor, BetI, was observed in vivo; in contrast, the *gbt* gene, required for choline and glycine betaine utilization as carbon source, was repressed in vivo indicating that GB is primarily used as osmo-protectant rather than for carbon and nitrogen acquisition.

### Energy production, carbon and micronutrients metabolism

The transcriptional profile of *P*. *aeruginosa* in sputum samples showed a marked shift towards a mixed anaerobic/micro-aerophilic respiration suggested by induction of the denitrification operons *nor*, *nir*, *nar*, *nos*, and *nap*. Furthermore, expression of terminal oxidases with high affinity to oxygen (*ccoOQP2*, *ccoN3*/PA14_40510), induced when oxygen tension is low (2% O_2_)^34,35^, was also increased in the lungs (Supplementary Data 2). A strong induction of a putative thiosulfate reductase-encoding operon (PA14_64540 - PA14_64560), indicates that thiosulfate might be an additional terminal electron acceptor during growth in mucus (Supplementary Data 2). No genes involved in pyruvate fermentation were affected, with the exception of the *adhA* gene, encoding an alcohol dehydrogenase, which was induced in vivo, whereas the formate dehydrogenase-encoding genes *fdnH* and *fdhE* genes were repressed (Supplementary Data 2).

**Fig. 4:**
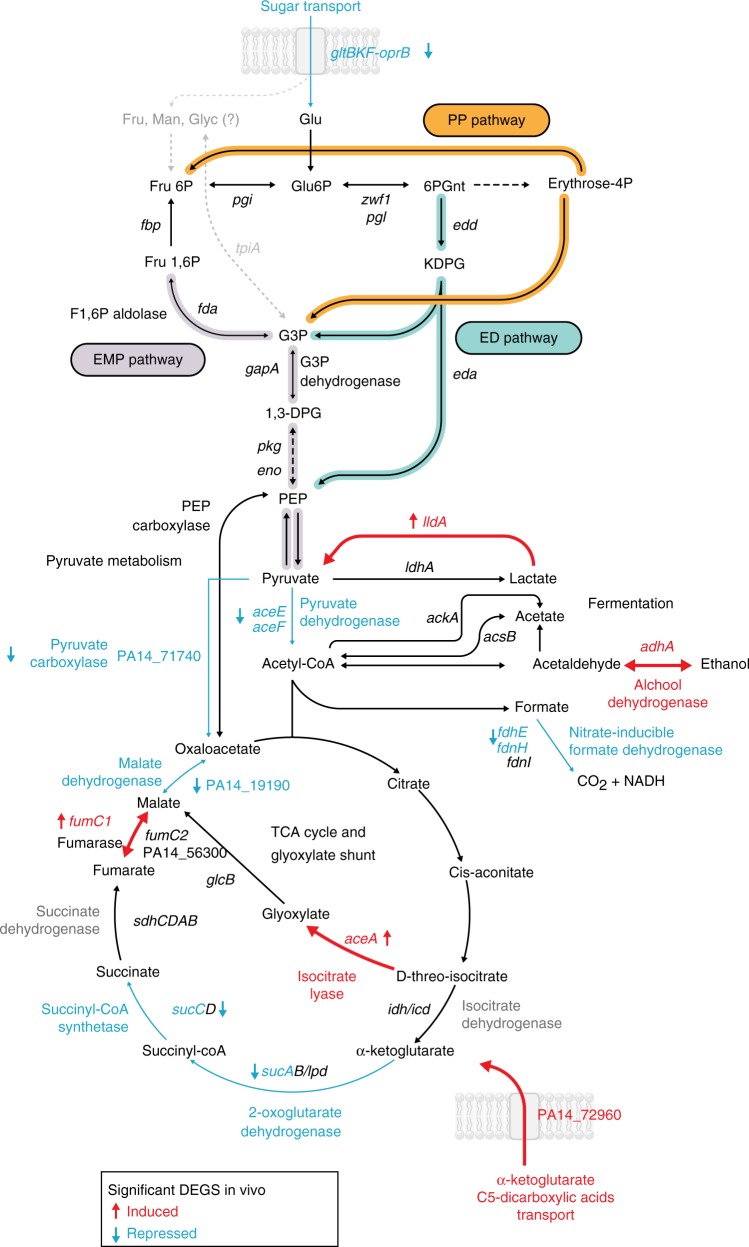
Effects of the CF lung environment on *Pseudomonas aeruginosa* central carbon metabolism. Significant changes of the transcriptomic pattern between the in vitro- and in vivo-grown bacteria are reported. Transcripts, encoded enzymes, and catalyzed reaction (arrows) enriched during growth in CF lung are given in red, and decreased transcripts are indicated in blue. Genes and reactions not statistically significant are reported in black. Dashed lines connecting metabolic intermediates indicate that the reaction is carried out by multiple consecutive enzymes not reported

By and large, we observed a general reduction of expression of genes involved in central carbon metabolism including those encoding for the sugar transporter *gltBFK-oprB* and for enzymes involved in pyruvate degradation to oxaloacetate (PA14_71740) and acetyl-CoA production from pyruvate (*aceEF*) (Fig. 4 and Supplementary Data 2). Similarly, expression of genes coding for enzymes of the lower part of the TCA cycle (*sucA, sucC*, and *lpd*) and of the hypothetical malate dehydrogenase PA14_19190 was reduced, while the transporter for the TCA intermediate α-ketoglutarate (PA14_72960) was stimulated in vivo. Increases in *aceA* expression suggest that isocitrate is preferentially channeled through the glyoxylate shunt and not funneled through decarboxylation steps of TCA cycle (Fig. 4 and Supplementary Data 2). We also observed a general induction in sputum samples of the *lldA* gene involved in L-lactate utilization, an abundant carbon source in CF sputum^36^.

Access to various restricted micronutrients represents another challenge for *P*. *aeruginosa* populations growing in the mucus layer. Indeed, more than 30% (*n* = 154) of the genes differentially expressed in vivo were related to acquisition of iron (*n* = 78), zinc (*n* = 33), sulfate (*n* = 36), and phosphate (*n* = 8) and were consistently stimulated in the lungs environment (Fig. 3b and Supplementary Data 2). Different genes involved in iron acquisition from heme (*phu*, *has* operons, and *hemO* and *hxuC* genes), as well as those involved in pyoverdine biosynthesis, secondary iron-scavanging systems from xeno-siderophores, and the ferric-citrate uptake regulators FecIR were specifically induced in the lungs (Supplementary Data 2).

**Fig. 5:**
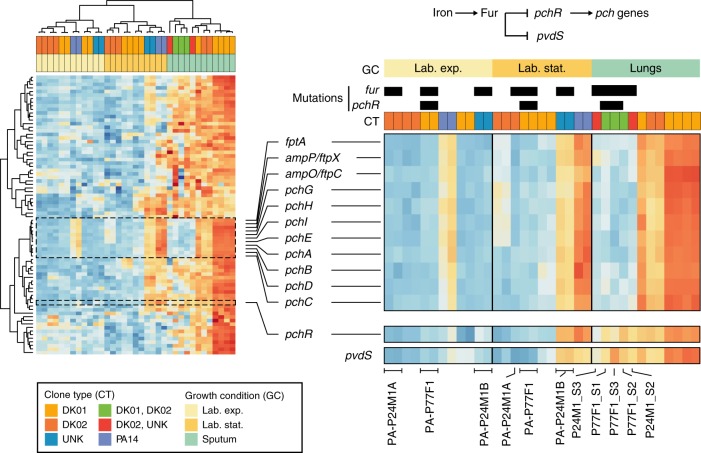
Transcriptional patterns of differentially expressed genes involved in iron acquisition and metabolism. Heat maps report the intensity of expression of each gene (rows) expressed as log-regularized read counts and scaled for each row. Each column represents a sample analyzed and is clustered based on the result of pvclust analysis. Growth conditions, clone type of each sample, presence of genetic mutations in *fur* and *pchR* genes (black bars), and a schematic representation of the regulation of pyochelin operon are reported

Interestingly, we noted that while significantly induced in the in vivo populations (Supplementary Data 2), the operons involved in pyochelin biosynthesis and uptake showed a strong polarization in expression levels in different in vivo subpopulations: transcript numbers where high in samples from patients P30M0 (DK01) and P73F1 (DK02), while low in those from P77F1 (DK01 and DK02) and P24M1 (DK02 and UNK) (Fig. 5). Expression of the pyochelin biosynthesis operon depends on the PchR activator in presence of iron together with the Fur regulator (Fig. 5). Although strains isolated from the respective expectorates (PA-P77F1 and PA-P24M1A/B) harbored non-synonymous mutations in the *fur* gene (Supplementary Data 3), Fur-dependent repression of the *pchR*, and *pvdS* genes was not eliminated. Indeed, expression of the two Fur-controlled genes was similar in strains with no mutations in the regulator (high in vivo/iron-limited and low in vitro/not limited) (Fig. 5). We identified a single non-synonymous mutation in the *pchR* coding sequences associated with strain PA-P77F1, but no mutations explaining the in vivo repression of *pch* operons were found in isolates from the patient P24M1. Thus, while genetic adaptation offers a simple explanation for the divergent in vivo expression observed for patient P77F1, different and less obvious genetic modifications, and/or responses to specific environmental conditions, might drive the reduction of *pch* expression in the expectorate from patient P24M1.

Similar to the iron acquisition system, we observed a strong induction of genes controlled by the Zur regulator and required in zinc-limiting conditions: putative zinc uptake systems, Zn^2+^-independent paralogs of metabolic enzymes (*pyrC2* and *folE2*), ribosomal proteins (*rpmE2* and *rpmJ*), and the stringent response mediator DskA2 (Supplementary Data 2). Induction of DskA2 in in vivo samples, was matched by repression of the zinc-dependent paralog DskA (Supplementary Data 2), indicating that stringent response is mostly driven by the Zn^2+^-independent DskA2 in the lungs.

Genes induced in sulfate-limiting conditions, in particular the *CysAWT-sbp* transporter and those involved in the acquisition of sulfur from organic compounds, including taurine and other organosulfonates were stimulated by CF airways environment (Supplementary Data 2). Additionally, a putative transporter implicated in sulfite/sulfoacetate excretion (PA14_27270) was induced in bacteria growing in mucus.

## Discussion

High-resolution sequencing of the total informative RNA species contained in expectorates of CF patients, provided an unprecedented insight into adaptive gene expression of *P*. *aeruginosa* in actual physio-pathological conditions. Although our observations are drawn from a limited number of samples, our sample set is more coherent, structured and larger than any previous study^37,38^. In particular, it comprises longitudinal samples from four patients with stable bacterial populations, representing gene expression variability in distinct clone types (inter-clonal diversity) and within the same clone type undergoing a within-patient independent evolution (intra-clonal diversity). It should be noted that, although gene expression from expectorates is a good predictor of the average physiology in different lung compartments^21^, our approach can only capture gene expression trends in the whole *P*. *aeruginosa* population and do not discriminate between phenotypically different sub-populations, including planktonic or aggregate cells, and other phenotypes such as small colony variants^39^. However, although providing limited disclosure of phenotypic and genetic heterogeneity, our approach has the effect of favoring a robust identification of strong global transcriptional patterns, which are shared across multiple clone types, and which represent the most relevant phenotypes of in vivo infections.

In our study we observe that the growth environment strongly impacts the transcriptional profiles of clinical isolates. In this sense, despite genetic differences deriving from independent accumulation of mutations in response to selective pressures operated in the lung environment for more than 30 years (Supplementary Table 4 and Supplementary Data 3), all lineages share similar transcriptional patterns in a specific environment (Fig. 2, lungs and laboratory conditions). Moreover, in laboratory conditions the transcriptional responses of clinical isolates deriving from the genes comprising the species soft-core genome (i.e., 5102 CDS) are similar to the one deriving from the reference strain PA14, which has not undergone to the same selective pressure of the lungs environment. This suggests that regulatory networks in clinical isolates are not strictly constrained by the mutations accumulated during the long-term adaptive process in the CF lungs environment. There are two interesting implications from these observations: (i) transcriptional plasticity, and thus ecological flexibility, is maintained regardless of a long adaptive specialization in the CF environment (although fitness in alternative environments most likely is reduced), and thus a broad spectrum of transcriptional responses may help preserving a high degree of genetic diversity despite strong selection pressures, favoring *P*. *aeruginosa* evolvability over strong specialization; (ii) assessments of phenotypic properties based on analysis of clinical isolates in laboratory conditions may strongly bias conclusions about in vivo bacterial physiology. This is particular relevant in clinical settings where a low correlation between antibiotic susceptibility testing and treatment outcome is often observed^40,41^. For example, we observe that in vivo expression of genes contributing to antibiotic tolerance is higher than in laboratory conditions, providing a simple explanation why antibiotic tolerance is often overlooked in the clinic.

Our analysis of bacterial in vivo gene expression in lungs of CF patients shows that common physiological functions can be recognized in heterogeneous populations. Indeed, based on the existence of a shared transcriptional program, we identified a core set of in vivo transcriptional phenotypes, which independent of the genetic background (Supplementary Table 1) represent acclimation to strong environmental factors in the lungs. We observed responses to oxidative and osmotic stress, antibiotic exposure, restricted access to metal ions, and DNA damage. The congruence between the observed phenotypes and the known conditions found in lungs^12,13^ underline the authenticity of the collected data.

Interestingly, we did not identify any preferred strategy to counteract stress or nutrient limitation. In fact, our data suggest that the infection success of *P*. *aeruginosa* reflects its ecological flexibility and the ability to differentially express multiple redundant pathways that exert similar biological functions^8^. For example, although we did observe a preference in iron acquisition from heme, as previously deduced from genetic data^42^, additional induction of the entire Fur regulon, and of other scavenging mechanism (Supplementary Data 2) may represent more optimal solutions for exploiting all the available iron sources. Interestingly, in vivo populations can show polarized expression of the pyochelin biosynthesis operon that seems to be relevant in the lungs but not for all populations (Fig. 5). Pyochelin has a lower affinity for iron than other siderophores, but it is more energy efficient, and is preferentially used when the concentration of iron is less limited^43^. We observed that differences in expression might be the result of single genetic mutations (patient P77F1) in the *pchR* gene, i.e., the most direct regulator of pyochelin biosynthesis, or either (1) be the result of more complex and less restrictive regulatory modifications or (2) potentially be dependent on specific environmental conditions (patient P24M1). This once more strengthens the idea that similar expression profiles could arise from genetically distinct populations, and that genetic heterogeneity is important for transcriptional plasticity allowing different populations to express the fittest phenotype in the appropriate condition.

We found the same physiological flexibility in the simultaneous stimulation of molecular pathways strictly required in anaerobic conditions and pathways expressed at low oxygen levels. This might reflect (i) the presence of defined transcriptionally active subpopulations inhabiting different niches (aerobic / anaerobic), or (ii) the concurrent expression of complementary systems that maximize the utilization of all the available electron acceptors, including oxygen, nitrogen and thiosulfate. Although our data cannot be used to distinguish between subpopulations, the lack of a strong stimulation of fermentative genes (Fig. 4 and Supplementary Data 2) might indicate that energy production requirement is fulfilled by the utilization of multiple respiratory acceptors in the large part of the population.

Reduced expression of genes involved in active growth (Fig. 3c and Supplementary Data 2) was accompanied by reduced expression of genes involved in central carbon metabolism and a redirection of carboxylic acids through the glyoxylate shunt with a net reduction of reducing potential. Rewiring of central carbon metabolism may reflect the availability of carbon sources. Although *P*. *aeruginosa* carbon preference is poorly understood in CF lungs, it is speculated that six carbon sources are primarily utilized in the mucus (proline, alanine, arginine, glutamate, aspartate, and lactate)^36^; with the exception of lactate dehydrogenase *lldA* gene, no other gene involved in degradation of these molecules was significantly increased in vivo. However, we did observe a strong repression of glucose transporter genes as well as genes involved in glycolysis and gluconeogenesis (Fig. 4 and Supplementary Data 2). Glucose is suggested to be present at milli-molar concentrations in CF secretions, but it is considered a secondary carbon source for *P*. *aeruginosa*^36^. We cannot exclude that the reduction of expression of genes in the central carbon metabolism is part of more complex adaptive phenotypes that cope with environmental stresses rather than reflecting any carbon source preference. Indeed, in vivo conditions promote expression of the *aceA* gene and a reduction of genes constituting the lower, NADH-producing, part of TCA cycle possibly resulting in an increased funneling of carbon atoms through the glyoxylate shunt. These metabolic modifications have been found to be important for protecting bacteria from antibiotics, and in general from ROS species^44–46^, and thus might play a central role for persistence in CF airways.

Additional carbon sources are available in CF lungs^36^. Surprisingly, we observed strong induction of genes involved in sulfate starvation response (Supplementary Data 2), although sulfate is not limited in the lungs^47^, and in fact is found at slightly higher concentration in CF sputum (~270 µM)^36^ than in LB medium (100–150 µM)^48^. In particular, growth in mucus stimulates expression of genes involved in uptake and degradation of organo-sulfonates. Induction of sulfate starvation related gene expression, despite non-limiting conditions, has been observed during *P*. *aeruginosa* infections in a plant model^49^, and was ascribed to host-dependent modulation of anion availability in the micro-environment surrounding the bacteria^49^. Importantly, the most abundant free organo-sulfonate, taurine, found in many tissues and in particular those under elevated levels of oxidative stress^50^ such as CF lungs, can be fermented^51^ or directly used as carbon and nitrogen source^52^. Accordingly, we observed that a gene encoding a hypothetical exporter involved in excretion of sulfite/sulfoacetate, a by-product of reactions involved in taurine utilization as nitrogen and carbon source, was induced in vivo. It is therefore tempting to speculate that induction of organo-sulfonate uptake and expression of sulfate-inducible genes in vivo is connected to C and N metabolism rather than being exclusively directed to sulfate acquisition. Similarly, *P. aeruginosa* clinical isolates can exploit mucins as sulfur source^47^, but additionally as carbon source after desulfurization, a mechanism that requires the activity of the glyoxylate shunt^53^. Further investigations are needed to validate additional contributions of potential carbon sources to *P. aeruginosa* survival in the lungs.

In summary, we provide a first detailed and organic gene-level representation of *P. aeruginosa* transcriptional phenotypes in CF lungs at a late stage of the infection. Our findings are not strongly restricted by genetic constraints and can therefore be considered as wide-spread strategies. This type of data may be considered potential platforms for identifying new therapeutic relevant targets in actual host environment, in particular for combined therapies, which could involve non-antibiotic molecules interfering with the in vivo physiology of the cell by modifying central carbon metabolism as recently suggested^46^. Nevertheless, further studies including a wider patient cohort and additional clone types are required to determine to what extent these patho-phenotypes are important for other clone types and in earlier stages of infection, when patho-adaptive mutations are limited, and transcriptional acclimation plays a predominant role.

## Methods

### Study cohort, samples collection, and processing

All sputum samples were collected at the Copenhagen Cystic Fibrosis Center at the University Hospital, Rigshospitalet, Denmark, from five chronically infected CF patients (median age 47.5 years, range 39.0–54.0 years) in stable condition visiting the outpatient clinic or hospitalized for routine intravenous antibiotic therapy (IV) (Fig. 1c and Supplementary Table 1). Each patient was characterized by different CFTR mutations, although all variations resulted in a severe phenotype (Supplementary Table 1). All patients were chronically infected with *P. aeruginosa* for more than 30 years (median years chronically infected 40.5, range 33.0–43.0 years), equivalent to ~200,000 generations^19^.

In order to stabilize RNA, and preserve the original transcriptome of the cellular communities, expectorates were collected directly from a patient and immediately added to 4 ml of freshly prepared sputum pre-lysis and preservation buffer (SLP buffer: 4 ml 1× DNA/RNA shield per 1–2 ml sputum sample, Zymo Research, USA; 200 mM Tris(2-carboxyethyl)phosphine, TCEP; 100 µg ml^−1^ Proteinase K) and vigorously shaken by hand until the samples were homogenous and completely lysed. Regardless of the volume and viscosity of the sample, the use of SLP buffer resulted in completion of the collection and pre-lysis step in less than 2 min. Stabilized samples were stored on ice and further processed on the same day or stored at −80 °C for no more than 2 days. To isolate *P. aeruginosa* bacteria for in vitro analysis, we collected a second sputum sample and isolated 10–100 single colonies of *P. aeruginosa* per sample by plating or streaking serial dilutions on selective media.

### Nucleic acid isolation from sputum samples

To extract total RNA we adapted the protocol by Lim and colleagues^37^ to our collection procedure. Briefly, pre-lysed samples stored in SLP buffer were transferred to a 15-ml centrifuge tube prefilled with 1 volume of Trizol® LS Reagent (Invitrogen) and 1 ml of Zirconium/glass beads (0.1 mm diameter, Carl Roth GmbH), and were bead-beaten on a horizontal shaker four times for one minute, in order to assure complete lysis of human and bacterial cells. After each iteration, the sample temperature was lowered by incubation of the tube on ice for 1 min. Samples in Trizol LS were briefly centrifuged to pellet beads, and the supernatant was split in multiple 1-ml aliquots to which 270 µl of chloroform was added. After shaking vigorously by hand for 15 s, samples were incubated for 2 min at room temperature and then centrifuged at 13,000 × *g* at 4 °C for 30 min to separate the aqueous phase from the phenol phase. All the RNA species longer then 17 nt were purified from the recovered aqueous phase using the RNA Clean & Concentrator™-25 (RCC) kit (Zymo Research), accordingly to the supplier’s protocol. After an initial quality control and quantification on Nanodrop (280/260 and 280/230 ratios were always higher than 1.9 and 2.2, respectively), 50–100 µg of total RNA were treated with 6–10 U of TURBO™ DNase (Invitrogen), and reactions were purified on-column using the RCC kit. As RNA extracted from sputum samples resulted always in partially degraded samples, to increase RNA quality and remove intrinsic background noise we size-selected the RNA recovering RNA species longer than 200 nt following the protocol supplied with the on-column purification kit. Recovered RNA was precisely quantified using fluorimetric quantitation with Qubit® RNA BR Assay kit (Invitrogen), and fragmentation state and RNA quality were assayed using RNA Nano kit on an Agilent Bioanalyzer 2100 machine (Agilent Technologies). All samples had an RNA integrity number (RIN) between 5 and 6, typical for partially fragmented samples. As RIN values from fragmented samples are not considered a sensitive measurement of RNA quality, we followed Illumina guidelines developed for formalin-fixed, paraffin-embedded (FFPE) samples measuring the percentage of RNA fragments >200 nucleotides (DV_200_). All samples included in this study had a DV_200_ ranging between 60 and 75% and were classified as medium high-quality samples according to Illumina prescriptions. When required, DNAase-treated samples were concentrated through ethanol precipitation.

### Strains, growth, and RNA extraction from laboratory cultures

The laboratory strain PA14 and the clinical strains PA-P30M0, PA-P24M1A, PA-P24M1B, PA-73F1, and PA-PA77F isolated from either the same expectorate used for in vivo RNA-seq analysis or from a second sputum sample collected concurrently (Supplementary Table 1), were grown in flasks in order to obtain reference transcriptomes representative of naïve and completely adapted strains. All strains were grown in LB medium at 37 °C in full aeration, achieved by shaking at 150 rpm per minute. Growth conditions were selected to barely resemble those typically found in the lungs^36,54^. Cells were harvested during mid-exponential and late stationary phases. Transcription was blocked adding 1 volume of cold stop solution (5% H_2_O-saturated phenol in ethanol) to 1 volume of bacterial culture in a pre-chilled collection tube. Supernatant was removed after centrifugation at 8000 × *g* at 4 °C for 10 min. Pellets were snap-frozen in an ethanol-dry-ice bath and stored at −80 °C for at least one night before proceeding to RNA extraction. Frozen samples were re-suspended in 200 µl 1× TE buffer containing 50 µg ml^−1^ Proteinase K and 100 µg ml^−1^ lysozyme. After 5-min incubation on ice, RNA was extracted from re-suspended pellets using Trizol LS reagent (750 µl) and purified from recovered aqueous phases using RCC columns (Zymo Research). Between 10 and 15 µg of total RNA were treated with 2 U of Turbo™ DNAse and the reactions were purified using RCC columns. RNA quality and integrity were assayed using RNA Nano kit on an Agilent Bioanalyzer 2100 machine. Samples with a RIN higher than 9 were used for preparing sequencing libraries.

### Library preparation and RNA sequencing

To evaluate the feasibility and sensitivity of the technique a pilot experiment was set up as follows: ~ 1 µg of total RNA of samples P30M0_S1, P24M1_S1, and P11F2_S1 were used as input to prepare strand-specific sequencing libraries using Illumina TruSeq mRNA kit. No rRNA-depletion was performed, and total RNA was directly added to fragment, prime, finish (FPF) mix solution. Sequencing libraries were prepared following the manufacturer’s instructions adjusting the fragmentation time to 6 min in order to cope with the partially fragmented nature of the samples. For all other samples used in this study, 300–500 ng of total RNA were depleted of ribosomal RNAs using RiboZero Epidemiology Gold kit (Epicentre). Samples enriched in mRNA species were used as input for preparing strand-specific libraries using ScriptSeq V2 kit (Epicentre) following the manufacturer’s instructions. To overcome the partially fragmented nature of the samples, the fragmentation step time was reduced after optimization to 3 min.

For transcriptional analysis of laboratory cultures ~500 ng of total RNA were used as input for the rRNA removal procedure and cDNA libraries were prepared from rRNA-depleted samples using ScriptSeq V2 kit following the standard protocol without additional modifications. After quality and size distribution check on DNA HS chips on an Agilent Bioanalyzer 2100 machine, libraries were pooled in equimolar amounts and sequenced on an Illumina NextSeq 500 machine. An average of 200 million 75-bp-long reads per sample, either single-end or paired-ends, were generated for cDNA libraries deriving from sputum samples, while sequencing libraries from laboratory cultures were sequenced to a depth of 15–20 million reads per sample (75 bp, paired-ends).

### Reads processing and mapping

For all RNA-seq experiments, low-quality bases and contaminant adapters were trimmed using Trimmomatic (v 0.35)^55^, discarding reads shorter than 35 nt (minimum length to avoid excessive human reads contamination in meta-transcriptomes). Reads were further processed using SortMeRNA tool (v 2.1)^56^ to remove reads generated from residual rRNA transcripts. For RNA-seq experiments deriving from pure cultures grown in laboratory conditions, processed reads were mapped against *P. aeruginosa* UCBPP-PA14 genome (NCBI: NC_008463.1) using BWA (0.7.15-r1140) aligner and MEM algorithm with defaults parameters. For meta-transcriptomes deriving from sputum sample, high-quality human and bacterial reads were separated in silico by mapping reads using BWA aligner and MEM algorithm against the human genome assembly GRCh38.p9 retrieved from NCBI database. All sequences aligning to human genome were discarded. Reads not mapping on the human genome were used as input for analyzing the transcriptionally active community, to evaluate interference of other bacteria to species-specific reads assignment and to asses *P*. *aeruginosa* gene expression in vivo.

### Pang-enome analysis and determination of core genome

Gene constituting *P. aeruginosa* pan-genome (Supplementary Data 1) were identified through pan-genome analysis using Roary tool with default parameters^57^.

Seventy-nine *P. aeruginosa* genomes marked as complete, including 63 deriving from human-associated pathogens and 11 from CF patients, were downloaded from NCBI assembly database and used as input for the tool. The complete list of RefSeq accession numbers is available in Supplementary Data 1. Genes conserved in 95% of the analyzed genomes (*n* = 75) were defined as species soft-core gene set (*n* = 5102), while genes conserved in 100% of the analyzed genomes (*n* = 2319) were defined as belonging to core gene set as previously suggested^58^.

### Gene expression analysis

For gene expression analysis, mapped reads deriving either from sputum samples or batch cultures were analyzed following the same procedure. Briefly, reads mapping on each annotated coding sequence of *P. aeruginosa* PA14 genome (NCBI, assembly GCF_000014625.1) were counted using htseq-count version 0.7.2^59^ and counts normalized using regularized log transformation performed using the rlog Transformation function contained in the R package DESeq2^60^ with option blind set as “True”. Normalized counts were used to evaluate whole transcriptome similarities using hierarchical clustering analysis (HCA), principal component analysis (PCA) and k-mean clustering on PCA-reduced data. HCA was performed using the function “pvclust” in the R package pvclust using Pearson’s correlation coefficient (1 – cor(), “cor” option) as a distance method and “ward.D2” as hclust method. Statistical support for clusters was obtained using multi-scale bootstrapping implemented in the same function, with the number of bootstraps set as 10,000 considering significant clusters with an Approximated Uncertainty (AU) *p* value ≤0.05. Sample correlation and clustering were visualized as dendrograms and heat maps using the R packages Dendextend and ComplexHeatmap. Principal component analysis on normalized reads counts was performed using prcomp() function with scale option set as “FALSE”. PCA-reduced data was clustered using k-means algorithm identifying the optimal number of clusters using the NBClust function contained in the NBClust package using all available indexes.

Differential gene expression (DEG) analysis between transcriptomes deriving from sputum samples and batch cultures was performed using the R package DESeq2, considering statistically significant genes with a Log_2_(FoldChange) ≥ |1.3| and an adjusted *p* value ≤0.05. DEGs were inspected and functional class enrichment was performed using the provided “term_erich.py” python script using gene-term associations present in COGs and KEGG classification databases obtained from Pseudomonas.com. Classes with a *p* value (Hyper-geometric test) and adjusted p-value (Bonferroni correction for multiple tests) ≤0.05 were considered statistically significant.

### Whole genome sequencing and genetics analysis

Genomic DNA was extracted using DNA Blood and tissue Kit (Qiagen) from *P. aeruginosa* isolated clones from sputum samples. Libraries were prepared using the NexteraXT kit (Illumina) and sequenced using a MiSeq machine with an approximate coverage of >50-fold. Reads were trimmed, and low quality reads and potential contamination from adapters were removed using Trimmomatic (v 0.35) tool^55^. Reads were mapped against the *P. aeruginosa* PA14 genome (NC_008463.1) using BWA MEM algorithm, and duplicated reads marked using Picard tools. GATK was used to re-align around microindels and to call variants using HaplotypeCaller algorithm (setting -ploidy 1)^61^. SNPs were extracted if they met the following criteria: a quality score of at least 50, a root-mean-square (RMS) mapping quality of at least 25 and a minimum of three reads covering the position. Microindels were filtered based on a quality score of at least 500, an RMS mapping quality of at least 25 and support from at least one-fifth of the covering reads. Variations unique to each clone belonging to the same lineage were used to determine potential transmissions and to estimate an average evolutionary distance expressed in years based on previously calculated within-patient mutation rate of 2.6 SNPs per year^2^ for normo-mutable strains and 100 SNPs per year for hypermutators^62^.

Determination of *P. aeruginosa* clone type from whole genome sequences was performed by comparing each clinical isolate genome sequence with those of *P. aeruginosa* clones isolated from other CF patients^2,6,7,22^. Clones that differed for less than 10,000 genetic variations were considered to belong to the same clone type. Assignation of clone types infecting each patient was either derived from whole genome sequencing obtained from representative isolates collected from patients’ sputum samples used in this study or previously published data^22^.

### Ethical approval and consent to participate

Use of the samples was approved by the local ethics committee at the Capital Region of Denmark Region Hovedstaden (registration number H-4-2015-FSP), and all patients gave informed consent. Transcriptional profiles deriving from human cells were discarded.

### Code availability

A thoroughly commented analysis of in vivo and in vitro gene expression, and code to generate the main figures and results of this work is available in the Zenodo repository (10.5281/zenodo.1162703). Access to the developed analysis pipeline deployed at Danish National Supercomputer is possible previous agreement with corresponding authors.

## Electronic supplementary material

Supplementary Information

Description of Additional Supplementary Files

Supplementary Data 1

Supplementary Data 2

Supplementary Data 3

## Data Availability

Raw sequence read data supporting the results of this work are available in the EMBL-EBI European Nucleotide Archive (ENA) under the Accession No. PRJEB24688. RNA-seq data deriving from sputum samples have been deprived of human transcripts to comply with rules approved by the local ethics committee at the Capital Region of Denmark Region Hovedstaden (registration number H-4-2015-FSP) on the usage of samples.
