# Supplementary material for: High-resolution in situ transcriptomics of Pseudomonas aeruginosa unveils genotype independent patho-phenotypes in cystic fibrosis lungs
Source: Nat Commun. 2018 Aug 27;9:3459. doi: 10.1038/s41467-018-05944-5 (PMC6110831; doi:10.1038/s41467-018-05944-5)
Supplement: Supplementary file 1 — Supplementary Information [file 41467_2018_5944_MOESM1_ESM.pdf]

High-resolution *in situ* transcriptomic of *Pseudomonas aeruginosa* unveils genetically-independent patho-phenotypes in cystic fibrosis lungs

Rossi *et al.*

Supplementary Note 1. Evaluation of transcriptionally active microbial community

Although patients included in this study were chronically infected with *P. aeruginosa*, the thick mucus layer also facilitates colonization with other opportunistic pathogens[1,2]. Therefore, we evaluated the relative composition of the transcriptionally active microbial community mapping reads identified as not assignable to the human genome against a curated database of clade-specific markers derived from diverse coding sequencing using the MetaPhlan 2 tool with default parameters[3]. The overall genera identified in all sputum samples are presented in Supplementary Figure 9. Although most genera were already associated with lungs microbiota of CF patients (Supplementary Figure 9), we noted that read assignment to operational taxonomic units (OUTs) of low abundance genera was not completely accurate and therefore excluded those representing less than 2% of the total population in all samples. A complete overview of the major transcriptionally active genera, thus excluding the filtered one, is presented in Supplementary Figure 2. Genera diversity within each sample is expressed using Shannon's diversity index, calculated using "diversity" function contained in R package "vegan".

Supplementary Note 2. Evaluation of transcriptional interference of co-infecting bacteria, and definition of read assignment to *P. aeruginosa*

Although cystic fibrosis patient included in this study were primary colonized with *P. aeruginosa* other bacterial species co-existed in patients' lungs (Supplementary Figure 2 and 9). In developing our analysis strategy, at first, we reasoned that reads originating from highly conserved regions in the genome of different bacteria can be erroneously assigned to *P. aeruginosa*, thus interfering with the correct quantification of gene expression for our target species. Therefore, we explored the potential bias introduced by mapping the non-human reads (NHR) directly against a single reference genome, i.e. *P. aeruginosa* strain UCBPP-PA14 (NCBI: NC_008463.1), and whether removal of ambiguous reads was necessary. At the same time, we also evaluated whether the use of multiple *P. aeruginosa* genomes could increase reads mapping coping with the potential genetic difference between different *P. aeruginosa* populations present in each patient. The employed workflow is summarized in Supplementary Figure 10. Briefly, for each sample, the total non-human reads (NHR) were mapped against the community pan-

genome (com_pangenome in Supplementary Figure 10), which was made up of all the complete genomes of species belonging to the genera representing at least 2% of the active community in at least one sample (see Supplementary Data 1 for complete list of accession numbers). Reads not mapping on community pan-genome were considered free of transcript deriving from bacteria other than *P. aeruginosa* and mapped against 79 deposited *P. aeruginosa* genomes marked as complete in NCBI database (pau_pangenome in Supplementary Figure 10. See Supplementary Data 1 for complete list of accession numbers). Reads mapping on "com_pangenome" were inspected using BLASTn to recover sequences that could be uniquely assigned to one of the *P. aeruginosa* genomes (identity > 95% on 99% of length) contained in "pau_pangenome". Reads reassigned to *P. aeruginosa* by BLASTn analysis were integrated with those mapping uniquely on "pau_pangenome" data set ("Filter and mapping on pan-genomes" strategy in Supplementary Figure 10) and compared with reads obtained by mapping directly ("Direct mapping" strategy in Supplementary Figure 10) the NHR datasets against *P. aeruginosa* PA14 genome. Reads shared between the two datasets were identified using reads unique identifiers (IDs).
When we used the "Direct mapping" strategy, a highly variable number of reads mapped directly on *P. aeruginosa* PA14 genome, with most of the samples (n = 10, ca. 77%) encompassing the range 1 – 5 million (Supplementary Table 3). As expected, the percentage of *P. aeruginosa* reads obtained agreed with the observed relative abundance of the pathogen in the transcriptionally active bacterial community, with samples dominated by *P. aeruginosa* (i.e. P30M0_S1, P30M0_S2, P24M1_S1, P77F1_S1, P11F2_S1) scoring the highest percentage of reads assigned to this bacterium (Supplementary Figure 2 and Supplementary Table 3). When we employed the "Filter and mapping on pan-genomes" strategy, on average, 85% reads (range 54% - 96%) were not assignable to the any bacterial species contained in the com_pangenome data set (Supplementary Table 3). Almost half of the sequences surviving the filtering step were assigned to *P. aeruginosa* by mapping against the pau_pangenome data set, showing figures similar to those obtained by directly mapping against the *P. aeruginosa* PA14 genome (Supplementary Table 3). Moreover, ~90% of the reads were shared between the two datasets in all samples. These figures increased to 99% when we reassigned the

sequences that mapped to the "com_pangenome" but that can be unequivocally assigned to only *P. aeruginosa* using BLASTn tool. Therefore, pre-filtering and mapping the filtered reads using a pan-genome of *P. aeruginosa* strains did not improved reads assignment compared to directly mapping total non-human reads against *P. aeruginosa* PA14 genome, and the faster direct method was chosen for our analysis. However, we anticipate that the negligible bias we observed is derived by the nature of our samples in which *P. aeruginosa* is the major pathogen. We therefore strongly advise reads filtering when the percentage of the target species drops under 10-15% of the total bacterial community, and the initial number of reads used for mapping is lower than 500,000 high-quality reads, as experienced with another analyzed sample not connected to this study.

Supplementary Note 3. Technical and biological reproducibility

In order to evaluate the technical reproducibility of the technique we used three samples (P30M0_S3, P30M0_S4 and P77F1_S1). Each sputum sample was physically split in two technical replicates at the time of collection and each replicate was processed independently, RNA sequenced, and gene expression evaluated as described in the Methods section. Normalized reads count and relative composition of the transcriptionally active microbial community were used to evaluate reproducibility calculated as Pearson correlation coefficient (r) (Supplementary Figure 4).

In order to evaluate the biological reproducibility of the technique we collected from the same patient two independent expectorates on the same day at a distance of 15 minutes (P30M0_S3, P30M0_S4) (Supplementary Table 1). Samples were processed independently, RNA sequenced, and gene expression evaluated as described in Methods section. Normalized reads count and relative composition of the transcriptionally active microbial community were used to evaluate reproducibility calculated as Pearson correlation coefficient (r) (Supplementary Figure 4).

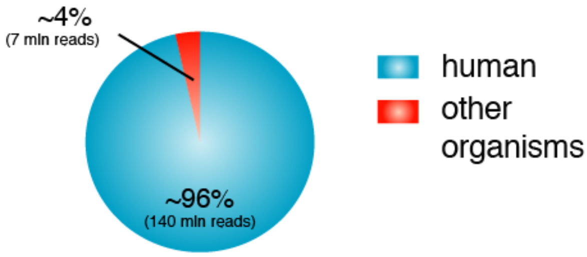

Supplementary Figure 1. Average reads composition obtained from sputum samples. The pie chart represents the average composition calculated on all samples included in this study. human: reads mapping on human genome assembly GRCh38.p9. other organisms: reads not mapping on human genome assembly GRCh38.p9.

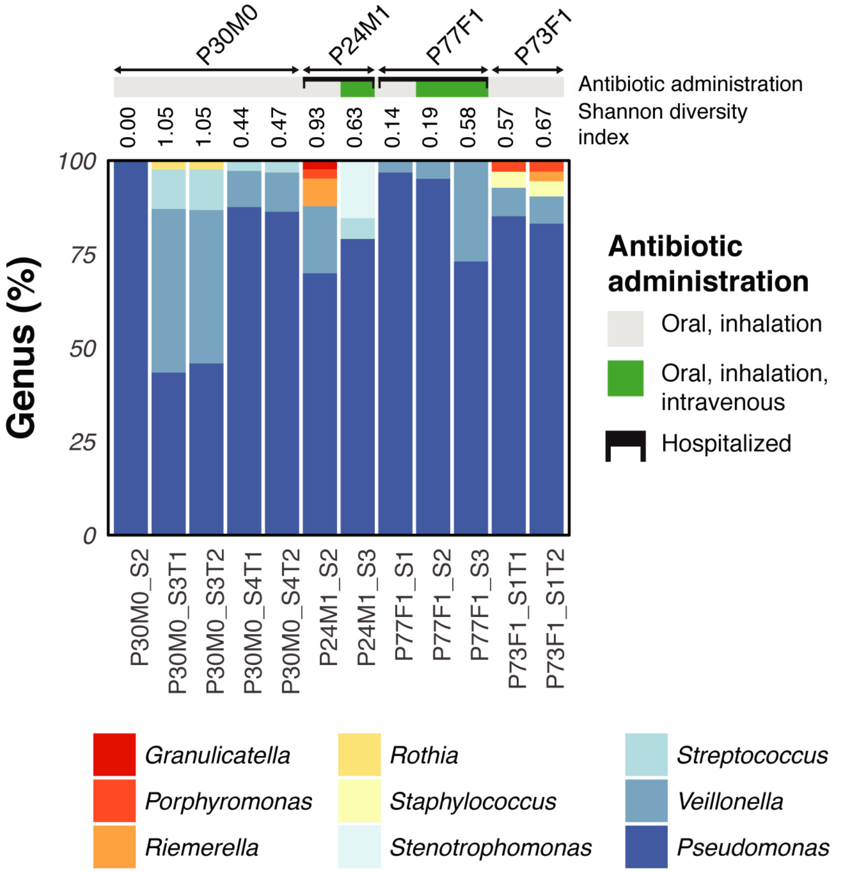

Supplementary Figure 2. Major transcriptionally active bacterial genera in sputum samples. Transcriptionally active genera identified using Metaphlan 2 tool. The graph reports only major genera passing the filtering criteria applied to raw data (see Supplementary Note 1). Intra-sample bacterial diversity is expressed using Shannon Diversity Index, with 0.0 representing the presence of only one dominant genus. Samples' and patients' identifiers, antibiotic administration (oral, inhalation, intravenous), and attendance to hospital are reported in the top meta-data bar.

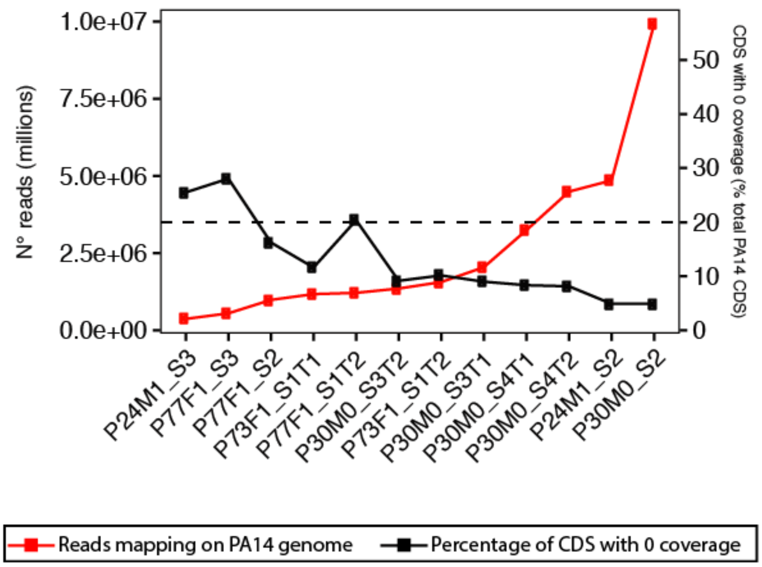

Supplementary Figure 3. CDS detection sensitivity. For each sample the plot represents the number of reads mapping on PA14 genome (red line and squares) and the corresponding percentage of coding sequences (CDS) with zero coverage (black line and squares) in each sample, calculated considering as total number of CDS the number of annotated genes in *Pseudomonas aeruginosa* PA14 genome (n = 5976).

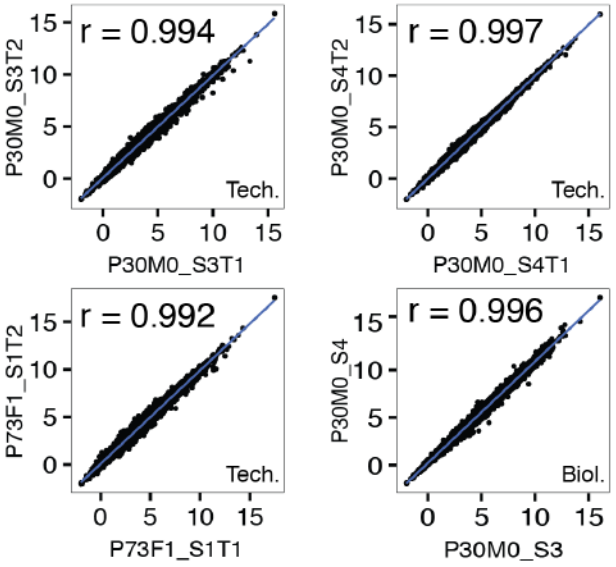

Supplementary Figure 4. Technical and biological reproducibility of the technique. Binary comparisons of gene expression (normalized gene counts) in technical (Tech.) and biological (Biol.) replicates of samples P30M0_S3, P30M0_S4, P73F1_S1. Biological replicates are defined as two independent sputum samples collected from the same patient on the same day. For biological replicates analysis (P30M0_S3, P30M0_S4) the average values deriving from two technical replicates (P30M0_S3T1/T2, P30M0_S4T1/T2) is plotted. Correlation between each replicates' pairs is reported as Pearson's correlation coefficient (r) for each set.

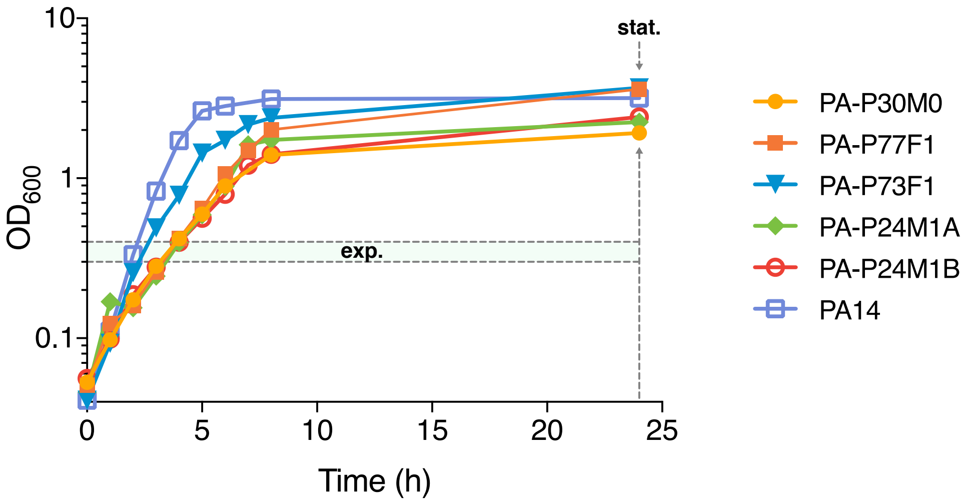

Supplementary Figure 5. Bacterial growth in laboratory conditions and sampling time. Standard laboratory strain *P. aeruginosa* PA14 and clinical isolates PA-P30M0, PA-P24M1A, PA-P24M1B, PA-P73F1, PA-P77F1 isolated respectively from P30M0_S2, P24M1_S2, P73F1_S1, P77F1_S1 sputum samples were grown in LB medium in flasks under aerobic conditions. During exponential (exp.) and stationary (stat.) phase samples were collected and gene expression level was quantified through RNA-seq.

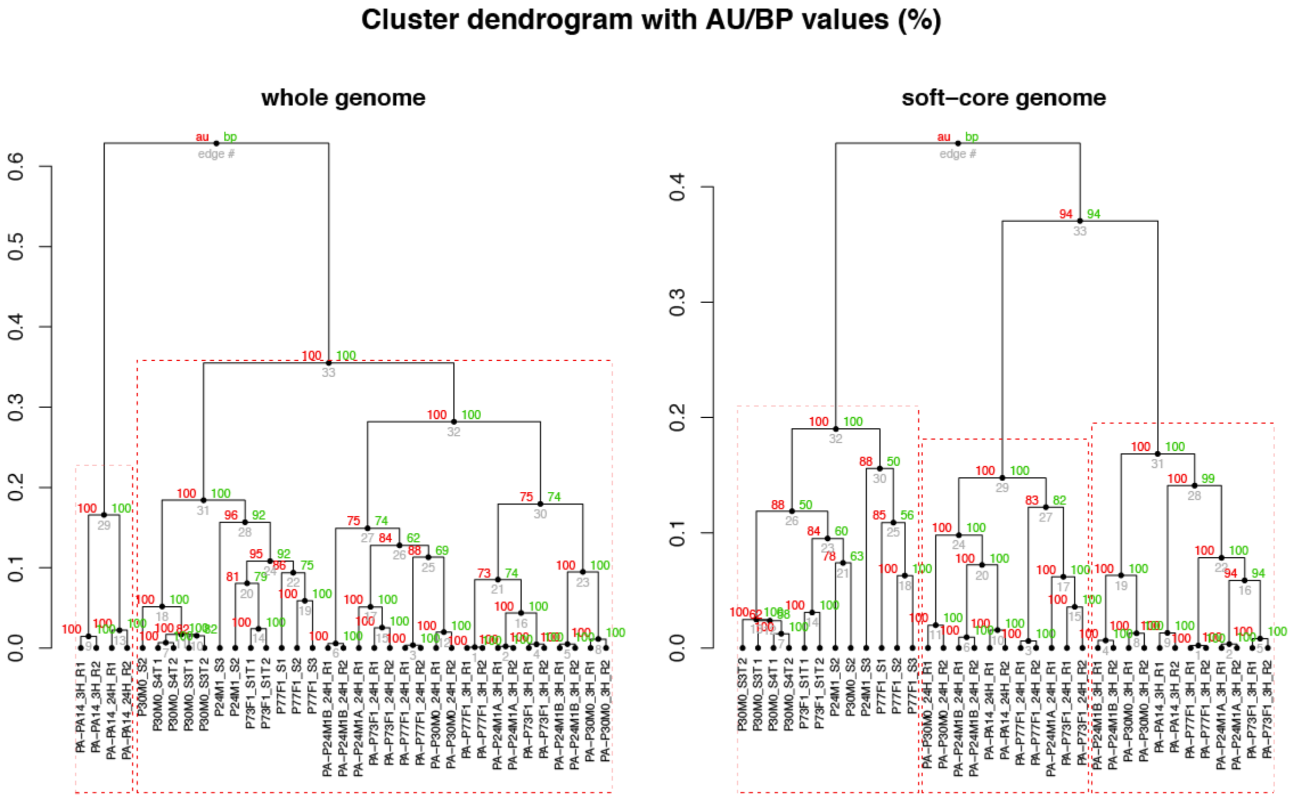

Supplementary Figure 6. pvclust analysis results. Dendrogram representing hierarchical clustering performed on correlation coefficients (Pearson's correlation coefficient) based on multi-scale bootstrap resampling (n = 10,000) implemented in pvclust R package (using Ward's method). Values on the edges represent Approximately Unbiased p-value (AU) and Bootstrap Probability (BP) values in percent. Primary clusters strongly supported by the data (AU > 95%, or significance level < 0.05) are highlighted by dashed red rectangles. Growth phases: 3H, exponential phase; 24H, stationary phase.

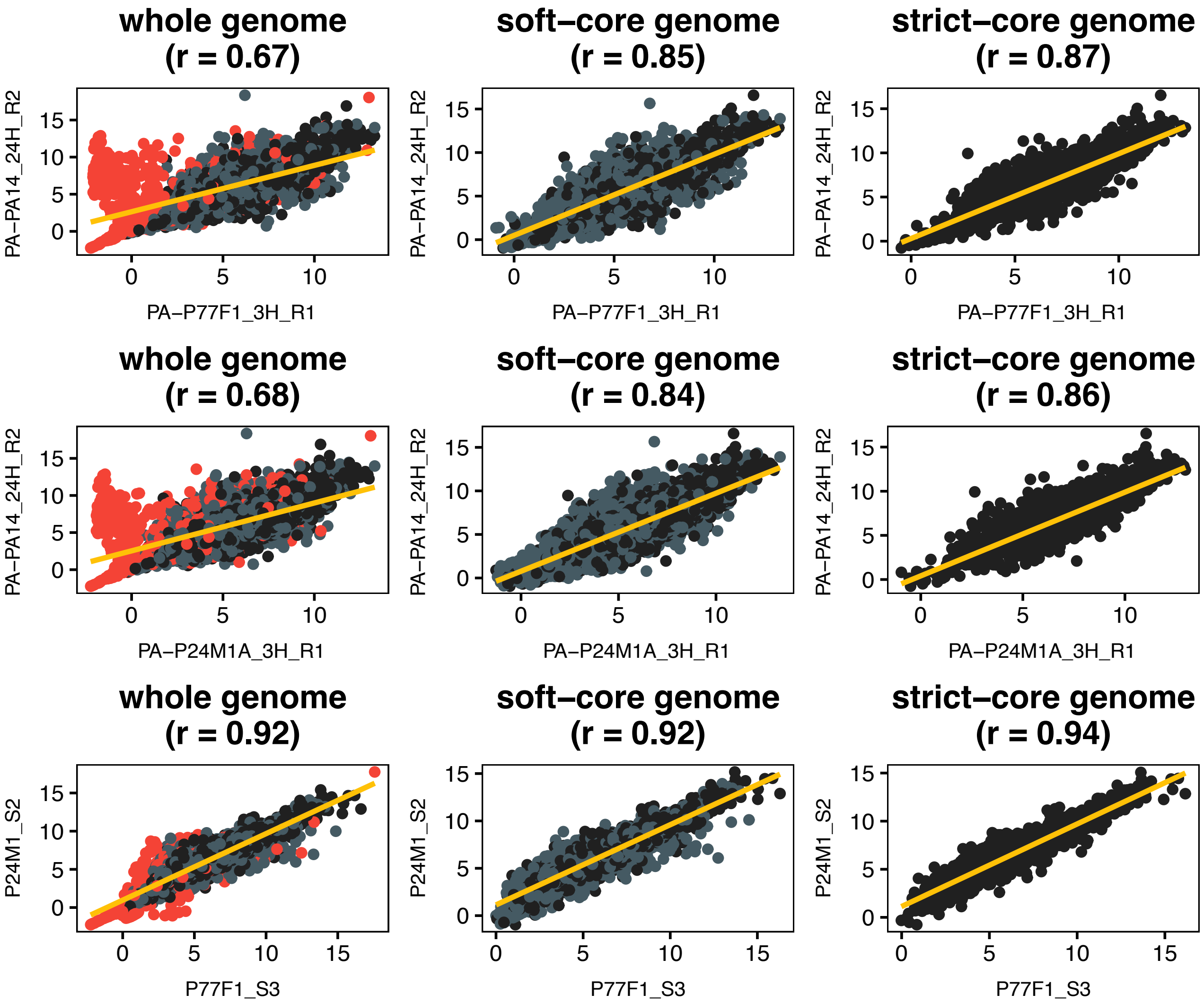

Supplementary Figure 7. Restricting gene expression analysis improve linearity between samples with low correlation due to genetic variation. Pairwise comparison of the normalized reads counts between samples with low (first two rows) and high (last row) Pearson's correlation coefficient (r). Linearity improves by removing source of noise due to genetic difference in samples with low correlation coefficient. Genes belonging to the accessory, soft-core and strict-core genomes are represented by red, gray and black dots, respectively.

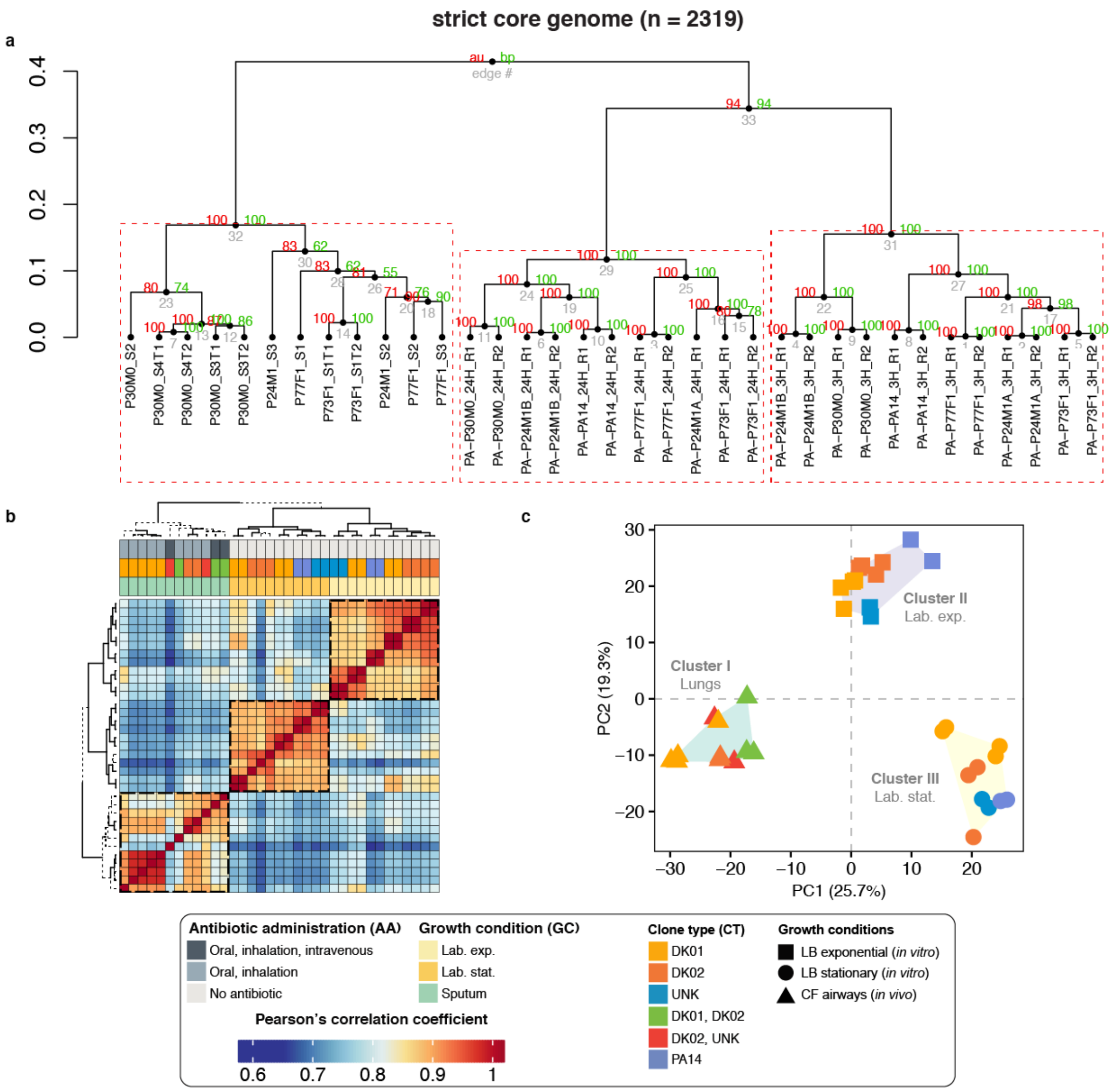

Supplementary Figure 8. Transcriptional profile similarities of genes comprising the core genome. a. Dendrogram representing hierarchical clustering performed on correlation coefficients (Pearson's correlation coefficient) based on multi-scale bootstrap resampling (n = 10,000) implemented in pvclust R package (using Ward's method). Values on the edges represent Approximately Unbiased p-value (AU, red) and Bootstrap Probability (BP, green) values in percent. Primary clusters strongly supported by the data (AU > 95%, or significance level < 0.05) are highlighted by dashed red rectangles. **b.** Gene expression correlation expressed as Pearson's correlation coefficient (r) and visualized as heatmap. Row and column clustering are based on results from pvclust analysis. Major significant clusters are highlighted by dashed rectangles, and solid lines in the side dendrograms. Dashed lines in dendrograms represents branches with AU values < 95% **c.** Cluster refinement by principal component analysis (PCA) and group identification based on k-means clustering on PCA data. Correlation coefficients and PCA analysis were performed on rLog-normalized counts, considering only coding sequences (CDS) conserved in the species core genome (n = 2319).

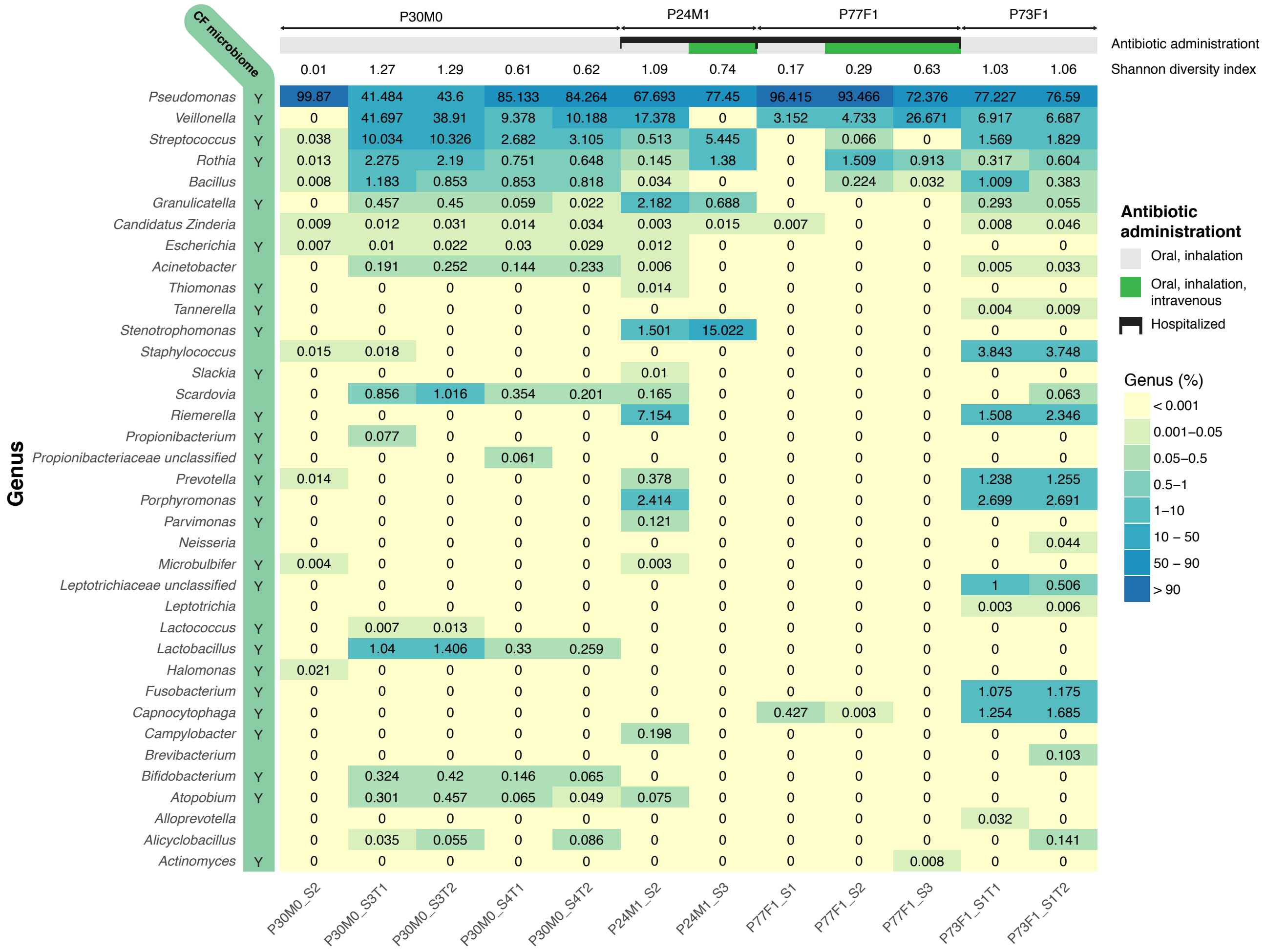

Supplementary Figure 9. Total transcriptionally active bacterial community in sputum samples. Relative abundance of bacterial genera identified by mapping total reads classified as non-human against a curated database of clade-specific markers. The graph report unfiltered data containing all the genera identified by MetaPhlan 2 tool. Samples' and patients' identifiers, antibiotic administration (oral, inhalation, intravenous), and attendance to hospital are reported. Bacterial genera diversity is measured using Shannon diversity index, with 0.0 representing the presence of only one dominant genus. If a genus was described in literature as associated with cystic fibrosis using either culture-dependent or culture-independent methods the "Y" character is reported in the CF microbiome column.

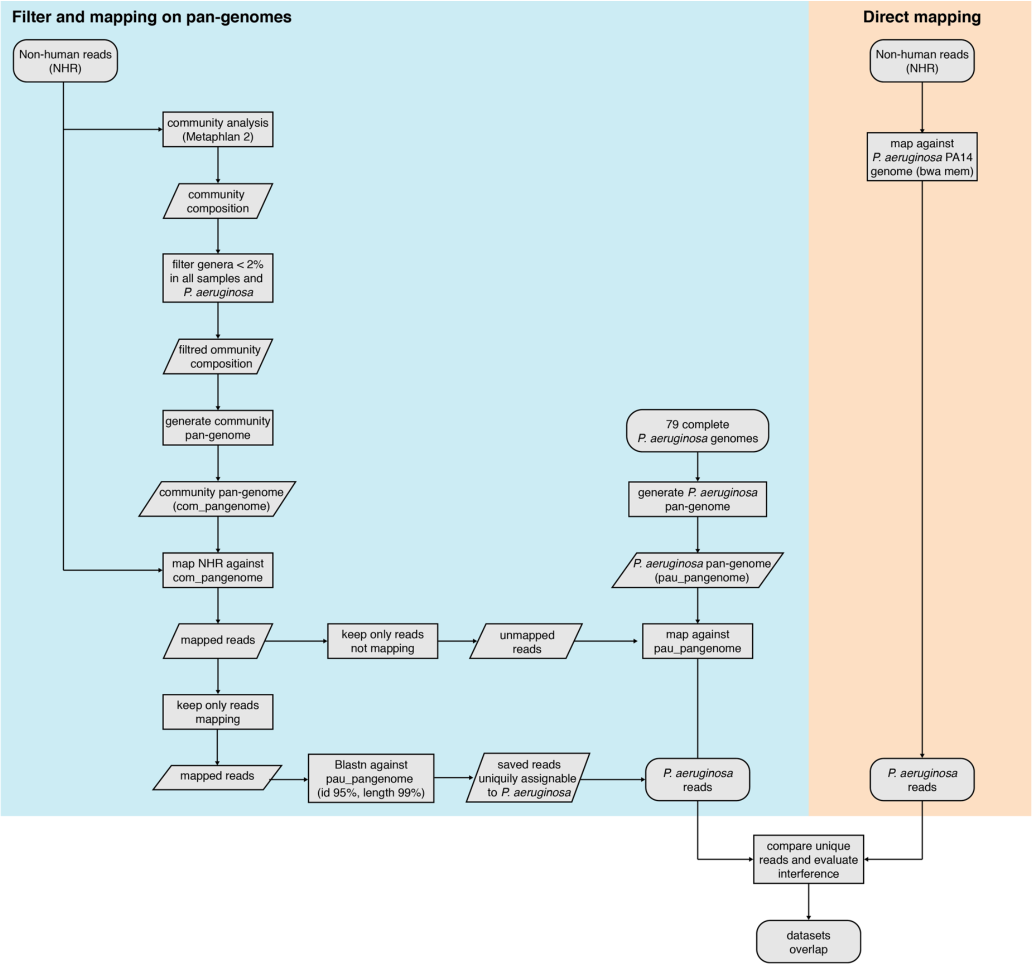

Supplementary Figure 10. Community effect on reads mapping. Flowchart representing the strategy employed to evaluate bias introduced in reads mapping by transcriptional activity of bacteria other than *P. aeruginosa* present in sputum samples included in this study.

Supplementary Table 1. Patients and sample information

Sample name	Collection date	Patient ID#	Patient genotype	Clone type*	Age	Chronic	Clinical status$	Clinical isolate (clone type)	Treatment§
P30M0_S1	25/05/16	P30M0	ΔF508/ W1282X	DK01	53	40	Stable	-	*IN*: aza, cst
P30M0_S2	23/06/16	P30M0		DK01	53	40	Stable	PA-P30M0 (DK01)	*IN*: aza, cst
P30M0_S3T1	28/02/17	P30M0		DK01	54	41	Stable	-	*IN*: aza, cst
P30M0_S3T2	28/02/17								
P30M0_S4T1	28/02/17	P30M0		DK01	54	41	Stable	-	*IN*: aza, cst
P30M0_S4T2	28/02/17								
P24M1_S1	16/06/16	P24M1	ΔF508/ ΔF508	DK02, UNK	52	43	Stable	-	*IV*: mem, tob / *OR*: caz
P24M1_S2	28/11/16	P24M1		DK02, UNK	52	43	Stable	PA-P24M1A (DK02) PA-P24M1B (UNK)	*IN*: caz
P24M1_S3	05/12/16	P24M1		DK02, UNK	52	43	Stable	-	*IN*: caz, tob/ *OR*: azm / *IV*: mem
P77F1_S1	18/11/16	P77F1	ΔF508/ 3659delC	DK01, DK02	43	40	Stable	PA-P77F1 (DK01)	*IN*: tob / *OR*: azm
P77F1_S2	25/11/16	P77F1		DK01, DK02	43	40	Stable	-	*IN*: tob / *IV*: c_t, cst
P77F1_S3	02/12/16	P77F1		DK01, DK02	43	40	Stable	-	*IN*: tob / *IV*: c_t, cst
P73F1_S1T1	22/02/17	P73F1	ΔF508/ ΔF508	DK02	42	33	Stable	PA-P73F1 (DK02)	*IN*: cst
P73F1_S1T2	22/02/17								
P11F2_S1	16/06/16	P11F2	ΔF508/ ΔF508	DK02	39	38	Stable	-	*IN*: cst / *OR*: cip / *IV*: ipm, tob

# Patient unique identifier reported in the form of P(AA)(S)(B). (AA)(B) combination represents numbers that uniquely identify each patient and (S) represents the sex of the patient: (M) male; (F) female.

* clone type was determined using whole genome sequences of clones isolated from the collected sputum samples and from previously published data[4].

$ we defined the health status of the patient as stable as no signals of exacerbation were detected.

§ **IN:** inhalation; **OR**: oral; **IV**: intravenous. aza: aztreonam (ß-lactam); azm: azithromycin (macrolide); caz: ceftazidime (ß-lactam); cip: ciprofloxacin (fluoroquinolone); cst: colistin (polymyxin); ipm: imipenem (carbapenem/ß-lactam); mem: meropenem (ß-lactam); tob: tobramycin (aminoglycoside); c_t: ceftolozane-tazobactam (ß-lactam).

Supplementary Table 2. Sequencing reads and alignment statistics

Sample name	Total reads*	Total non-ribosomal reads (NRR)		Total human reads#		Total non-human reads$	
		n° reads	% total	n° reads	% of NRR	n° reads	% of NRR
Pilot study (rRNA not depleted)							
P30M0_S1	180,282,389	58,431,088	32.41%	57,098,909	97.72%	1,332,179	2.28%
P24M1_S1	195,782,357	66,055,720	33.74%	64,218,293	97.22%	1,837,427	2.78%
P11F2_S1	180,502,739	40,973,181	22.70%	36,598,295	89.32%	4,374,886	10.68%
Study samples (rRNA depleted)							
P30M0_S2	191,000,058	185,227,908	96.98%	174,265,080	94.08%	11,521,760	6.22%
P30M0_S3T1	147,966,660	144,459,615	97.63%	136,236,857	94.31%	8,222,758	5.69%
P30M0_S3T2	150,975,307	148,725,102	98.51%	143,101,985	96.22%	5,623,117	3.78%
P30M0_S4T1	195,296,346	149,116,247	76.35%	142,086,791	95.29%	7,029,456	4.71%
P30M0_S4T2	126,959,180	124,542,734	98.10%	116,714,186	93.71%	7,828,548	6.29%
P24M1_S2	156,136,726	153,431,325	98.27%	133,984,686	87.33%	19,446,639	12.67%
P24M1_S3	125,012,936	122,869,676	98.29%	119,858,742	97.55%	3,010,934	2.45%
P77F1_S1	247,876,696	203,160,331	81.96%	201,398,786	99.13%	1,761,545	0.87%
P77F1_S2	150,376,851	135,711,786	90.25%	133,622,193	98.46%	2,089,593	1.54%
P77F1_S3	154,635,295	130,384,367	84.32%	128,305,785	98.41%	2,078,582	1.59%
P73F1_S1T1	97,600,776	93,563,222	95.86%	90,371,088	96.59%	3,192,134	3.41%
P73F1_S1T2	162,723,163	156,753,888	96.33%	152,270,284	97.14%	4,483,604	2.86%

* total numbers of reads passing quality control
# total numbers of reads mapping on human genome assembly GRCh38.p9
$ total numbers of reads not mapping on human genome assembly GRCh38.p9

Supplementary Table 3. Evaluation of transcriptionally active microbial community to *Pseudomonas aeruginosa* transcripts assignation

Sample name	Non-human reads (NHR)	Reads mapping on PA14 genome (MPA14)		Reads not mapping on community pan-genome# (NCPR)		NCPR mapping on *P. aeruginosa* pan-genome$			Total reads assignable to *P. aeruginosa*‡	
		n° reads	% total NHR	n° reads	% total NHR	n° reads	% total NCPR	% id. MPA14*	n° reads	% id. MPA14*
Pilot study (rRNA not depleted)										
P30M0_S1	1,332,179	893,569	67.1%	1,275,242	96%	849,427	67%	94%	892,397	99.9%
P24M1_S1	1,837,427	884,989	48.2%	1,770,430	96%	865,438	49%	93%	882,440	99.7%
P11F2_S1	4,374,886	2,761,650	63.1%	4,194,402	96%	2,653,129	63%	94%	2,760,499	99.9%
Study samples (rRNA depleted)										
P30M0_S2	11,521,760	9,913,486	86.0%	10,202,586	89%	8,828,022	87%	87%	9,875,826	99.6%
P30M0_S3T1	8,222,758	2,041,465	24.8%	7,056,562	86%	2,002,618	28%	95%	2,034,677	99.7%
P30M0_S3T2	5,623,117	1,350,735	24.0%	4,881,284	87%	1,323,914	27%	95%	1,346,359	99.7%
P30M0_S4T1	7,029,456	3,241,785	46.1%	6,727,028	96%	3,164,451	47%	95%	3,234,290	99.8%
P30M0_S4T2	7,828,548	4,490,615	57.4%	7,394,817	94%	4,363,476	59%	95%	4,479,855	99.8%
P24M1_S2	19,446,639	4,853,442	25.0%	10,455,484	54%	4,498,910	43%	88%	4,794,408	98.8%
P24M1_S3	3,010,934	367,308	12.2%	2,657,826	88%	343,735	13%	90%	359,804	98.0%
P77F1_S1	1,761,545	1,211,407	68.8%	1,526,516	87%	1,154,741	76%	92%	1,199,292	99.0%
P77F1_S2	2,089,593	982,681	47.0%	1,913,837	92%	950,423	50%	91%	979,762	99.7%
P77F1_S3	2,078,582	546,962	26.3%	1,192,375	57%	521,514	44%	91%	545,113	99.7%
P73F1_S1T1	3,192,134	1,169,239	36.6%	2,765,423	87%	1,083,161	39%	88%	1,166,316	99.8%
P73F1_S1T2	4,483,604	1,560,508	34.8%	3,833,369	85%	1,408,906	37%	86%	1,556,518	99.8%

community pan-genome: all genomes marked as completed at the time of the study deposited in NCBI genome database of species belonging to genera that comprise more than 2% of the active transcriptional community in at least one sample. See Supplementary Data 1 for a complete list.

$ *P. aeruginosa* pan-genome: 79 *P. aeruginosa* genomes marked as completed at the time of the study deposited in NCBI genome database. See Supplementary Data 1 for a complete list.

* % id. MPA14: Fraction of reads mapping on *P. aeruginosa* pan-genome – with or without BLASTn-mediate reassignment of filtered reads – contained in the reads pool mapping on *P. aeruginosa* PA14 genome. Common reads were identified by the unique identifier assigned during sequencing.

‡ Total reads assignable to *P. aeruginosa* are defined as all the reads uniquely mapping on *P. aeruginosa* pan-genome after removing reads mapping on community pan-genome, plus all reads mapping on community pan-genome that can be uniquely reassigned to *P. aeruginosa* strains using BLASTn tool.

Supplementary Table 4. Genetic distance (number of SNPs and microindels) between isolates of the same clone type

**DK01 clone type**		
	**PA-P77F1**	
	n° variations*	Est. evolutionary years#
**PA-P30M0**	6,149	61 – 2,365 years
**DK02 clone type**		
	**PA-P73F1**	
	n° variations*	Est. evolutionary years#
**PA-P24M1A**	5,237	52 – 2,000 years

* Total number of unique variations (SNPs and microindels) that separate two strains belonging to the same clone type.

Estimated evolutionary years separating two isolates based on previously calculated within-patient mutation rate of 2.6 SNPs per year (top limit, normo-mutable[5,6]) or 100 SNPs per year (bottom limit, hypermutators[7])

Supplementary References

1. Bittar, F. & Rolain, J.-M. Detection and accurate identification of new or emerging bacteria in cystic fibrosis patients. *Clin. Microbiol. Infect.* **16,** 809–820 (2010).
2. Coburn, B. *et al.* Lung microbiota across age and disease stage in cystic fibrosis. *Sci. Rep.* **5,** 10241 (2015).
3. Truong, D. T. *et al.* MetaPhlAn2 for enhanced metagenomic taxonomic profiling. *Nat. Meth.* **12,** 902–903 (2015).
4. Jelsbak, L. *et al.* Molecular epidemiology and dynamics of *Pseudomonas aeruginosa* populations in lungs of cystic fibrosis patients. *Infect. Immun.* **75,** 2214–2224 (2007).
5. Marvig, R. L., Johansen, H. K., Molin, S. & Jelsbak, L. Genome analysis of a transmissible lineage of *Pseudomonas aeruginosa* reveals pathoadaptive mutations and distinct evolutionary paths of hypermutators. *PLoS Genet.* **9,** e1003741 (2013).
6. Marvig, R. L., Sommer, L. M., Molin, S. & Johansen, H. K. Convergent evolution and adaptation of *Pseudomonas aeruginosa* within patients with cystic fibrosis. *Nat. Genet.* **47,** 57–64 (2015).
7. Feliziani, S. *et al.* Coexistence and within-host evolution of diversified lineages of hypermutable *Pseudomonas aeruginosa* in long-term cystic fibrosis infections. *PLoS Genet.* **10,** e1004651 (2014).
