# Supplementary material for: High-resolution in situ transcriptomics of Pseudomonas aeruginosa unveils genotype independent patho-phenotypes in cystic fibrosis lungs
Source: Nat Commun. 2018 Aug 27;9:3459. doi: 10.1038/s41467-018-05944-5 (PMC6110831; doi:10.1038/s41467-018-05944-5)
Supplement: Supplementary file 2 — Description of Additional Supplementary Files [file 41467_2018_5944_MOESM2_ESM.docx]

**Description of Additional Supplementary Files**

File Name: Supplementary Data 1

Description: *P. aeruginosa* pan-genome analysis: strains and genes.

File Name: Supplementary Data 2

Description: *In vivo* differentially expressed genes.

File Name: Supplementary Data 3

Description: Common and unique SNPs and microindels identified in clinical isolates.
